# Neuron-targeted overexpression of caveolin-1 alleviates diabetes-associated cognitive dysfunction via regulating mitochondrial fission-mitophagy axis

**DOI:** 10.1186/s12964-023-01328-5

**Published:** 2023-12-15

**Authors:** Wenxin Tang, Chaoying Yan, Shuxuan He, Mengyu Du, Bo Cheng, Bin Deng, Shan Zhu, Yansong Li, Qiang Wang

**Affiliations:** 1 Department of Anesthesiology & Center for Brain Science, The First Affiliated Hospital of Xi’an Jiaotong University, Xi’an, 710061 Shanxi China; 2 Department of Anesthesiology and Perioperative Medicine, The First Affiliated Hospital of Nanjing Medical University, Nanjing, 210029 China

**Keywords:** Caveolin-1, Mitochondrial fission, Mitophagy, Diabetes-associated cognitive dysfunction

## Abstract

**Background:**

Type 2 diabetes mellitus (T2DM) induced diabetes-associated cognitive dysfunction (DACD) that seriously affects the self-management of T2DM patients, is currently one of the most severe T2DM-associated complications, but the mechanistic basis remains unclear. Mitochondria are highly dynamic organelles, whose function refers to a broad spectrum of features such as mitochondrial dynamics, mitophagy and so on. Mitochondrial abnormalities have emerged as key determinants for cognitive function, the relationship between DACD and mitochondria is not well understood.

**Methods:**

Here, we explored the underlying mechanism of mitochondrial dysfunction of T2DM mice and HT22 cells treated with high glucose/palmitic acid (HG/Pal) focusing on the mitochondrial fission-mitophagy axis with drug injection, western blotting, Immunofluorescence, and electron microscopy. We further explored the potential role of caveolin-1 (cav-1) in T2DM induced mitochondrial dysfunction and synaptic alteration through viral transduction.

**Results:**

As previously reported, T2DM condition significantly prompted hippocampal mitochondrial fission, whereas mitophagy was blocked rather than increasing, which was accompanied by dysfunctional mitochondria and impaired neuronal function. By contrast, Mdivi-1 (mitochondrial division inhibitor) and urolithin A (mitophagy activator) ameliorated mitochondrial and neuronal function and thereafter lead to cognitive improvement by inhibiting excessive mitochondrial fission and giving rise to mitophagy, respectively. We have previously shown that cav-1 can significantly improve DACD by inhibiting ferroptosis. Here, we further demonstrated that cav-1 could not only inhibit mitochondrial fission via the interaction with GSK3β to modulate Drp1 pathway, but also rescue mitophagy through interacting with AMPK to activate PINK1/Parkin and ULK1-dependent signlings.

**Conclusions:**

Overall, our data for the first time point to a mitochondrial fission-mitophagy axis as a driver of neuronal dysfunction in a phenotype that was exaggerated by T2DM, and the protective role of cav-1 in DACD.

**Graphical Abstract:**

Graphic Summary Illustration. In T2DM, excessive mitochondrial fission and impaired mitophagy conspire to an altered mitochondrial morphology and mitochondrial dysfunction, with a consequent neuronal damage, overall suggesting an unbalanced mitochondrial fission-mitophagy axis. Upon cav-1 overexpression, GSK3β and AMPK are phosphorylated respectively to activate Drp1 and mitophagy-related pathways (PINK1 and ULKI), ultimately inhibits mitochondrial fission and enhances mitophagy. In the meantime, the mitochondrial morphology and neuronal function are rescued, indicating the protective role of cav-1 on mitochondrial fission-mitophagy axis.

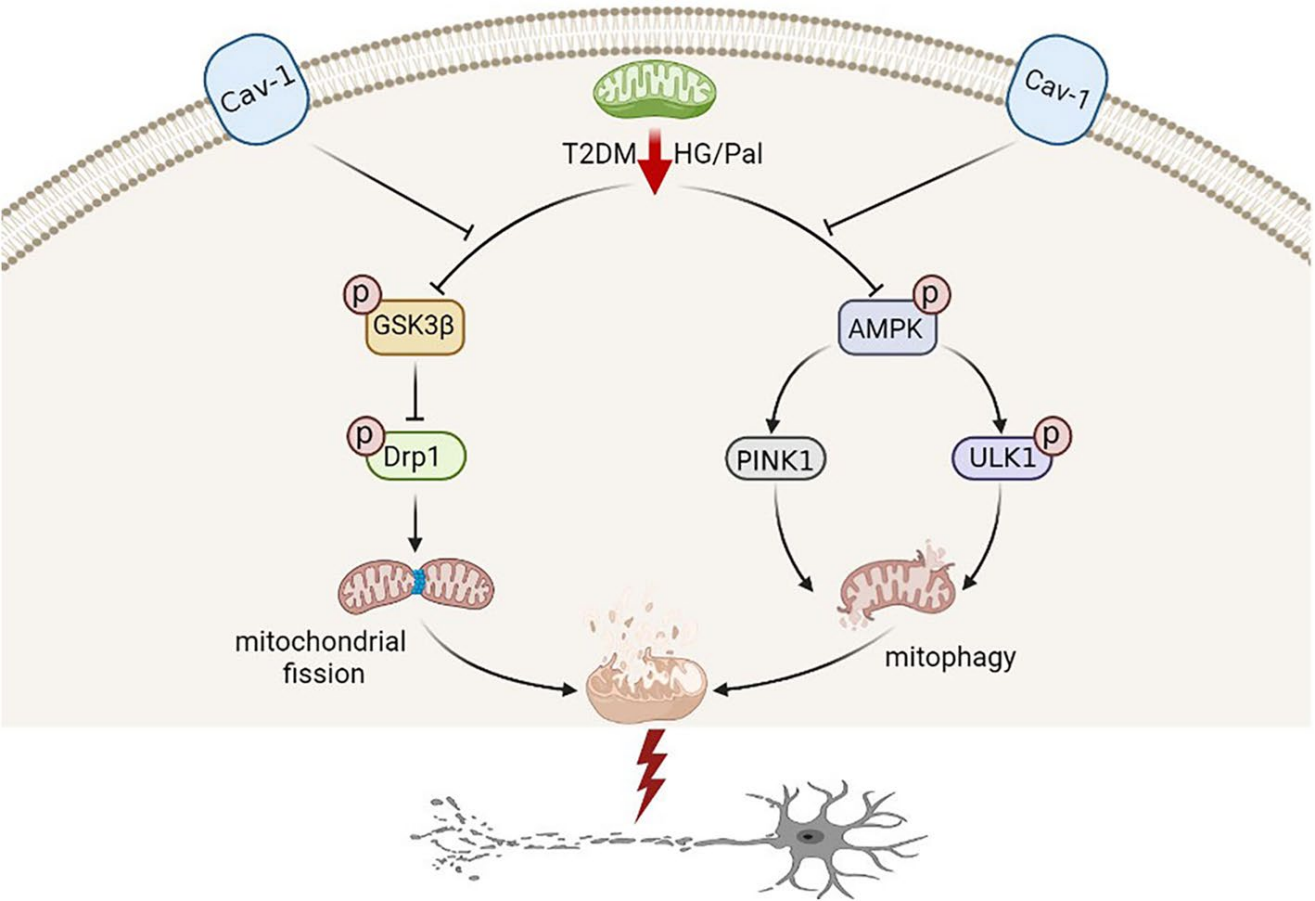

Video Abstract

**Supplementary Information:**

The online version contains supplementary material available at 10.1186/s12964-023-01328-5.

## Introduction

Type 2 diabetes mellitus (T2DM) was one of the well-established independent risk factors for dementia [1], which has been individually associated with both accelerated cognitive decline and an increased risk of cognitive impairment in several epidemiological studies [2–4]. T2DM and its progression is associated with a 1.5 to 2.5-fold increased risk for dementia, and this trend is similar for both Alzheimer’s disease and Parkinson’s Disease [5, 6], which are the most common subtypes of dementia. In 2021, 537 million adults worldwide were living with T2DM, and this is expected to increase to 783 million by 2045, with a 49% increase [7]. T2DM induces diabetes-associated cognitive dysfunction (DACD) imposes a significant health burden due to the increasing number of individuals afflicted with T2DM, and the resulting pathological consequences on cognitive processes. In this sense, it is urgent to dissect the cellular and molecular mechanisms underlying DACD.

Mitochondria are crucial metabolic hubs that generate ATP essential for neuronal growth, function, and regeneration via oxidative phosphorylation (OXPHOS) [8]. Compelling pathological and genetic data define mitochondria failure as the cause of numerous acquired diseases, especially neurodegeneration [9]. Mitochondrial quality control is mediated by mitochondrial dynamic processes, coupled with continuous cycles of fission and fusion [10]. Fusion and fission events work together to maintain the crucial cellular functions, including mitochondrial respiratory activity, mitochondrial DNA distribution, ATP production, apoptosis, cell survival or calcium signaling [11]. Of note, mitochondrial fission appears to go in-hand with mitophagy. Normally, damaged mitochondria undergo asymmetrical fission, which generates small and isolated mitochondrial fragments of feasible size, followed by targeted encapsulation via autophagosome [12]. Both of mitochondrial fission and mitophagy have been widely reported to be implicated in T2DM and its various complications [11, 13, 14].

In particular, accumulating evidence supports that excessive mitochondrial fission pave the way for DACD [15–17], which is a multistep process mainly depended on dynamin-related protein-1 (Drp1) [18]. In addition, phosphorylation of Ser^616^ on Drp1 was demonstrated to be a critical step enabling Drp1 GTPase activity for mitochondrial fission, which could be directly inhibited by p-ser^9^ glycogen synthase kinase 3β (GSK3β) [15, 19]. Also a point of contention is mitophagy, the specialized form of autophagy, serves as a primary mitochondrial quality control machinery to selectively encapsulate and clear damaged mitochondria [20]. In mammals, there are both PTEN-induced kinase 1 (PINK1)/Parkin-dependent pathway and PINK1/Parkin-independent pathways of mitophagy [21]. Numerous clinical and experimental studies have implicated a potential correlation between deficient mitophagy and DACD [22, 23]. Up to date, however, there are few studies investigating the specific role and mechanism of mitochondrial fission-mitophagy axis in the DACD, which still needs more exploration and tangible evidence.

Compelling pathological and genetic data defines caveolin-1 (cav-1) as a versatile membrane lipid raft scaffolding protein, raises the essence of cav-1 in various cellular activities [24, 25]. Earlier, we summarized a comprehensive overview about how cav-1 modulates pathogeneses of neurodegenerative diseases [24]. Subsequently, we further confirmed that cav-1 is a novel candidate with anti-oxidative and neuroprotective effects in DACD through modulating neuronal ferroptosis-mediated mitochondrial homeostasis [26]. Yet, it is still not clear whether cav-1 could improve DACD by modulating mitochondrial fission-mitophagy axis.

Therefore, in this study, we set out to explore mitochondrial fission-mitophagy axis in the occurrence and development of DACD. Understanding hippocampal neuronal specific responses to T2DM condition will aid in our understanding of precise cues for DACD. As we expected, T2DM environment significantly prompted mitochondrial fission, whereas mitophagy was blocked rather than boosted, which was accompanied by dysfunctional mitochondria and impaired neuronal function. Furthermore, we proved cav-1 improved T2DM-induced DACD by inhibiting excessive mitochondrial fission and stimulating defective mitophagy to balance the mitochondrial fission-mitophagy axis.

## Results

### T2DM induces excessive mitochondrial fission and mitochondrial dysfunction

**Fig. 1:**
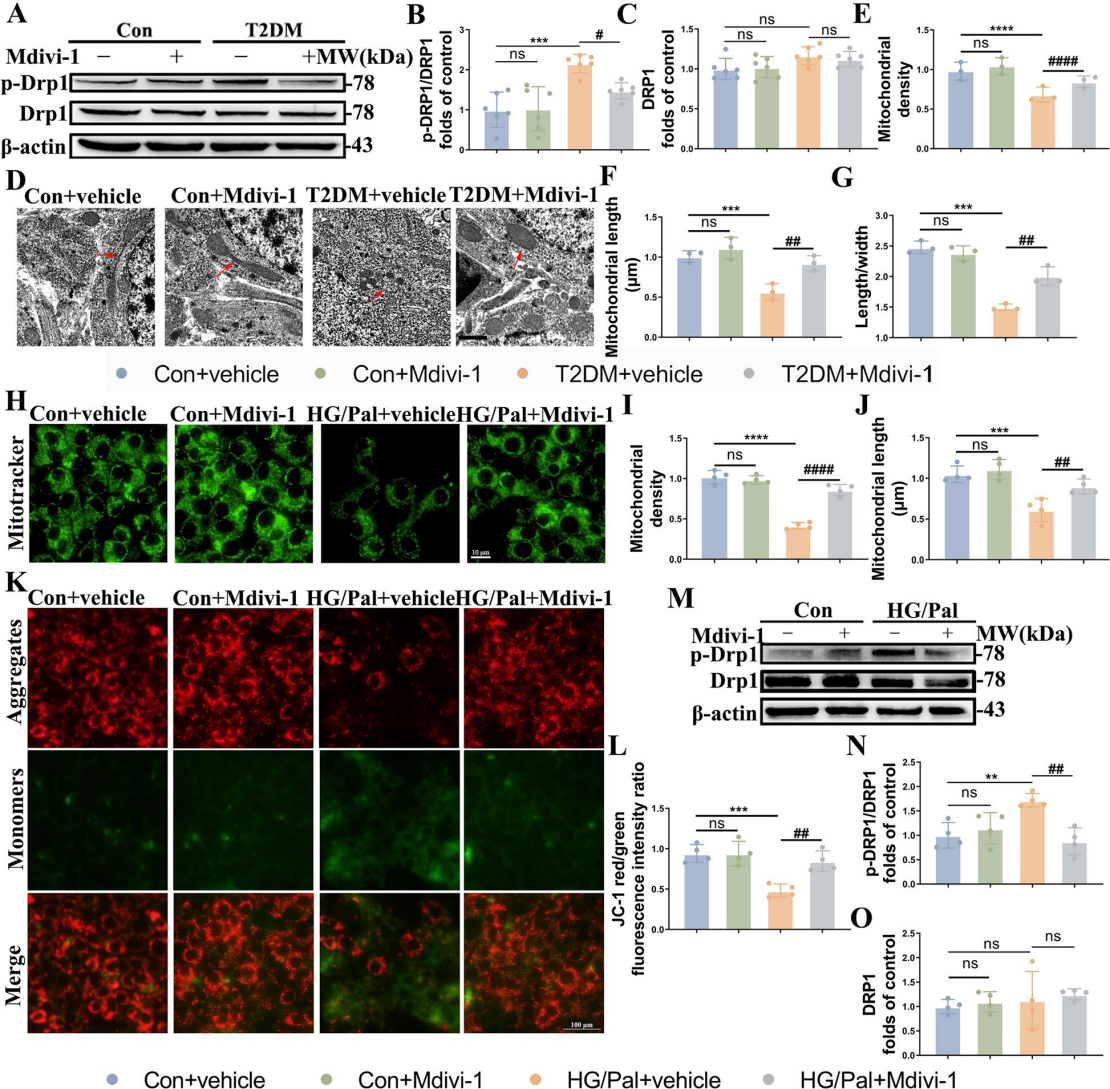
Mitochondria fission is increased in the T2DM environment. **A**-**C** Representative Western blot and densitometric analysis of p-Drp1/Drp1 and Drp1 in the hippocampal tissue lysates (*n* = 6). **D-G** Transmission electron microscopy showing mitochondrial morphology of neurons in the hippocampus of different groups, and measurements of mitochondrial density (**E**), length (**F**), ratio of length to width (**G**) (*n* = 3). Scale bar: 1 µm. **H-J** Representative Mito-Tracker and densitometric analysis of mitochondrial density and length in HT22 cells from the different groups (*n* = 4). Scale bar: 10 µm. **K**, **L** Representative JC-1 staining images and analysis in HT22 cells of different groups (*n* = 4). Red and green showed JC-1 aggregates and monomers respectively. Scale bar: 100 µm. **M–O** Representative Western blot and densitometric analysis of p-Drp1/Drp1 and Drp1 in the lysates of HT22 cells (*n* = 4). The results are represented as mean ± SD. ^*^P < 0.05 versus Control + vehicle, ^#^*P* < 0.05 versus T2DM + vehicle group, ^**^P or ^##^*P* < 0.01, ^***^P or ^###^*P* < 0.001, ^****^P or.^####^*P* < 0.0001

It has been shown previously that mitochondrial abnormalities in the hippocampus of db/db mice are induced by increased mitochondrial fission via a GSK3β/Drp1-dependent mechanism [15]. Our study validated the above results using T2DM mice and HT22 cells with HG/Pal. We found significantly increased phosphorylation of Ser616 on Drp1 in the T2DM group (Fig. 1A-C), accompanied by marked alterations in mitochondrial morphology (Fig. 1D). Also, intracellular smaller and ruptured mitochondria, accompanied with decreased mitochondrial density and length (Fig. 1E, F), as well as the ratio of mitochondrial length/width (Fig. 1G) in the CA1 neurons of T2DM mice were observed by transmission electron microscope (TEM), which were characterized as mitochondrial morphological features of mitochondrial fission. As expected, the above mitochondrial division-associated changes in the T2DM model were restored by the mitochondrial fission inhibitor Mdivi-1.

Next, we validated the above-described results in the T2DM mice in vitro. We treated HT22 cells with HG/Pal and Mdivi-1. The results of Mito-Tracker and JC-1 staining suggested that HG/Pal significantly reduced mitochondrial density and length (Fig. 1H-J), as well as mitochondrial membrane potential (MMP), which were recovered following Mdivi-1 treatment (Fig. 1K, L). In addition, HT22 cells exposed to HG/Pal presented much higher level of pDrp1/Drp1 compared to untreated HT22 cells, which was reversed by Mdivi-1 (Fig. 1M-O). Our data verified that T2DM model promotes hippocampal neuronal excessive mitochondrial fission.

### Mdivi-1 ameliorates DACD by improving mitochondrial and neuronal function in T2DM mice

**Fig. 2:**
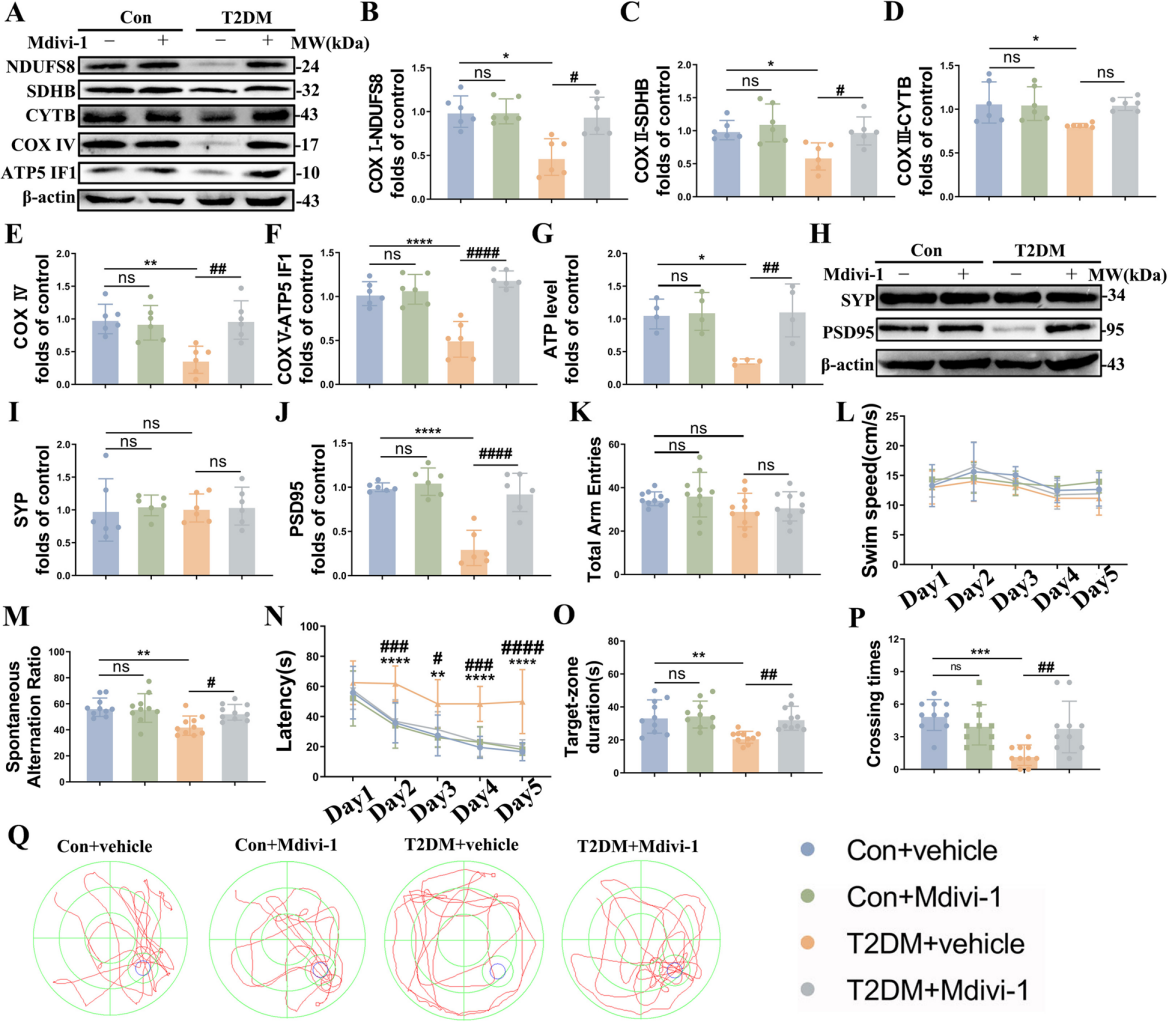
Inhibition of mitochondria fission by Mdivi-1 improves mitochondrial and neuronal function in HFD/STZ mice. **A-F** Representative Western blot and densitometric analysis of OXPHOS-related proteins, including complex I (NDUFS8), complex II (SDHB), complex III (CYTB), complex IV and complex V (ATPase IF1) in the hippocampal tissue lysates (*n* = 6). β-actin is used as a loading control. **G** ATP level in the hippocampus (*n* = 4). **H-J** Representative Western blot and densitometric analysis of PSD95 and SYP levels in the hippocampal tissue lysates (*n* = 6). β-actin is used as a loading control. **K**, **M** Total arm entries (left) and spontaneous alteration ratio (right) in the Y-maze test (*n* = 10). **L**, **N-P** The Morris water maze (MWM) analysis is quantified to obtain the (**L**) swimming speed, (**N**) latency, (**O**) crossing times, and (**P**) target-zone duration (*n* = 10). **Q** Representative traces from the MWM test. The results are represented as mean ± SD. ^*^P < 0.05 versus Control + vehicle, ^#^*P* < 0.05 versus T2DM + vehicle group, ^**^P or ^##^*P* < 0.01, ^***^P or ^###^*P* < 0.001, ^****^P or ^####^*P* < 0.0001

Given that the importance of mitochondrial morphology and function in synaptic function, we then further examined the expressions of the mitochondrial OXPHOS proteins (complex I-V) and ATP level to evaluate the mitochondrial function. Compared to the control model, the expressions of complex I-V and ATP concentration were dramatically decreased in the T2DM mice, while Mdivi-1 reversed this process (Fig. 2A-G). Of interest, the physiological and metabolic parameters (body weight, blood glucose, serum fasting insulin and HOMA-IR levels) of T2DM + vehicle and T2DM + Mdivi-1 groups were comparable, which indicated that Mdivi-1 did not exert effects by altering the physiological metabolism mentioned above (Fig S2 A-D). Furthermore, we quantified synaptophysin (SYP) and PSD95 to evaluate synapse function. Consistent with our previous findings [27], there was no significant difference in the level of SYP among all groups, except for an apparent decreased PSD95 expression in the T2DM mice, which was rescued following the Mdivi-1 administration (Fig. 2H-J).

Subsequently, we performed the Y maze and MWM tests to assess spatial learning memory and long-term spatial memory, respectively. There were no differences in locomotion among the four groups (Fig. 2K, L). During the Y maze test, the T2DM model exhibited a much lower spontaneous alternation rate as demonstrated previously, which again was reversed effectively by Mdivi-1 (Fig. 2M). Furthermore, in the MWM test, the T2DM mice showed a prolonged latency to reach the hidden platform in the acquisition phase of the MWM, while Mdivi-1 showed protective effects on cognition of the T2DM mice (Fig. 2N). In the probe test, compared with T2DM group, Mdivi-1 efficiently increased the number of platform crosses and prolonged time spent in the target quadrant (Fig. 2O-Q). Taken together, the results of behavioral tests demonstrated Mdivi-1 administration could markedly attenuate worsened cognitive ability in the T2DM mice.

### T2DM promotes decreased mitophagy and mitochondrial dysfunction in the hippocampus

**Fig. 3:**
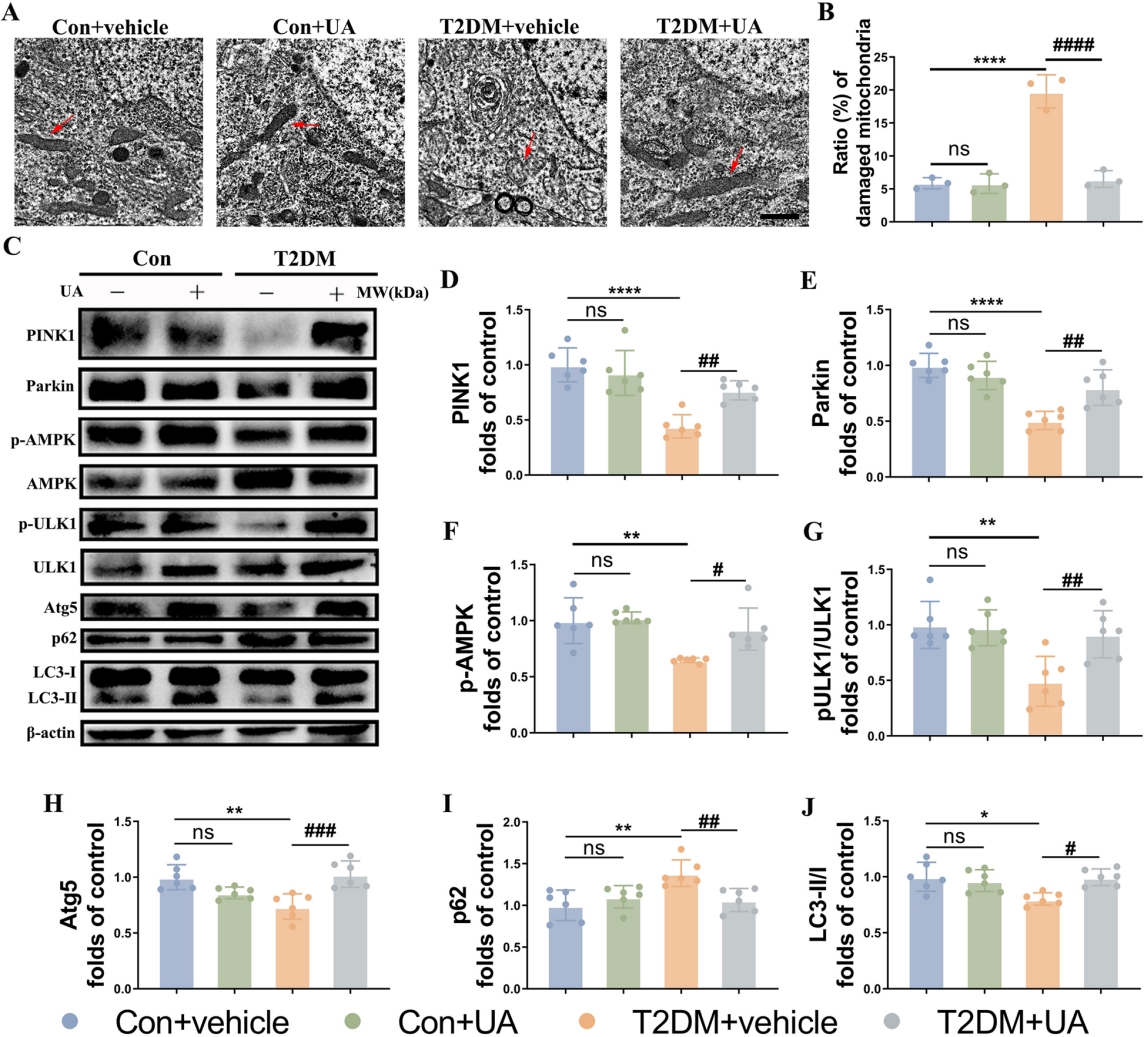
Mitophagy is decreased in the hippocampus of T2DM mice. **A** Transmission electron microscopy showing mitochondrial morphology of neurons in the hippocampus of different groups, and ratio of damaged mitochondria (*n* = 3). **C-J** Representative Western blot and densitometric analysis of PINK1, Parkin, p-AMPK/AMPK, p-ULK1/ULK1, Atg5, p62 and ratio of LC3II/I in the hippocampal tissue lysates (*n* = 6). β-actin is used as a loading control. The results are represented as mean ± SD. ^*^P < 0.05 versus Control + vehicle, ^#^*P* < 0.05 versus T2DM + vehicle group, ^**^P or ^##^*P* < 0.01, ^***^P or ^###^*P* < 0.001, ^****^P or.^####^*P* < 0.0001

There is now some evidence that mitophagy is involved in the process of DACD [22, 23], however, direct evidence is still lacking and the exact mechanism remains unclear. As shown in Fig. 3, the results of TEM showed that the mitochondria swelled and shortened, and the mitochondrial cristae disappeared in the T2DM group, accompanied with higher ratio of damaged mitochondria (Fig. 3A, B). Further, to confirm that mitophagy is also responsible for DACD induced by T2DM, we examined the AMP activated protein kinase (AMPK, the crucial activator of PINK1/Parkin and ULK1 pathway [28–32]), PINK1/Parkin pathway (conventional mitophagy) and ULK1 (Atg1/Unc-51 like autophagy activating kinase 1)-dependent (PINK1/Parkin-independent) pathway to assess mitophagy [28, 29, 33]. Compared with the control group, the phosphorylation level of AMPK at tyrosine 172, the expressions of PINK1, Parkin, autophagy protein 5 (Atg5), the phosphorylation level of ULK1 at Ser555 and the ratio of LC3II/I were significantly decreased, while p62 expression was elevated in the T2DM mice (Fig. 3C-I). Considering that AMPK is a downstream effector of UA (mitophagy activator) [34, 35], we think that UA induced the PINK1/Parkin and ULK1 pathway, and partially normalized the mitochondrial morphology through AMPK (Fig. 3C-I).

To confirm the above results, we further performed in vitro studies in HG/Pal treated HT22 cells. A tendency was observed in western blotting results of cells that was in parallel to the results in vivo (Fig S3 A-H). Meanwhile, we subjected HT22 cells to mitophagy detection, which revealed that cellular mitophagy was impeded by HG/Pal, but returned to normal following UA treatment (Fig S3 I, J). Consistent with the mitophagy detection, the results of JC‐1 staining showed UA effectively stabilized the MMP of HG/Pal treated HT22 cells (Fig S3 K, L). Overall, these data suggested T2DM inhibits mitophagy in the hippocampal neurons.

### UA improves DACD by improving mitochondrial and neuronal function in T2DM

**Fig. 4:**
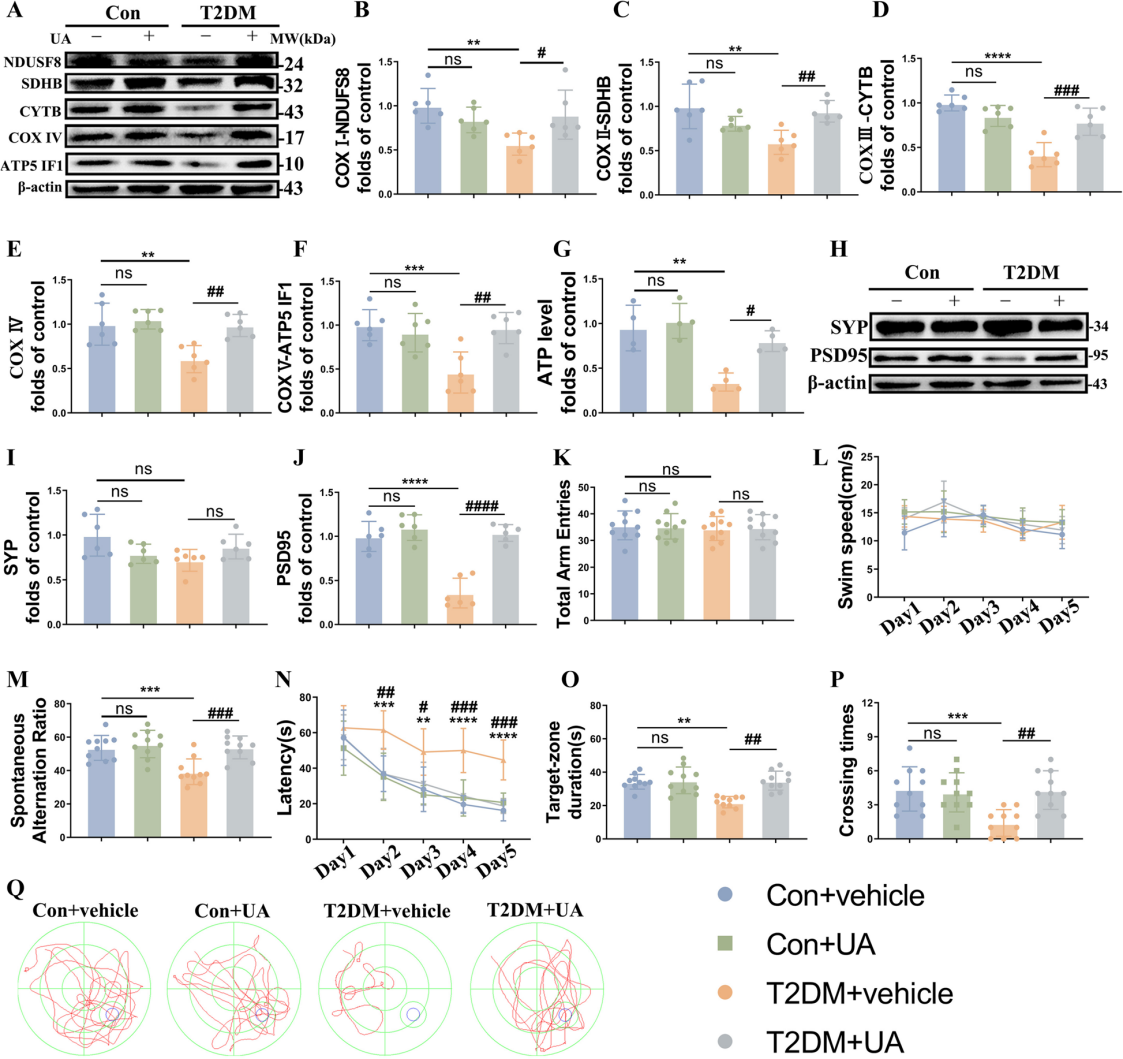
Enhancement of mitophagy by UA relieves mitochondrial and neuronal injury and cognitive dysfunction of T2DM mice. **A**-**F** Representative Western blot and densitometric analysis of OXPHOS-related proteins, including complex I (NDUFS8), complex II (SDHB), complex III (CYTB), complex IV and complex V (ATPase IF1) in the hippocampal tissue lysates (*n* = 6). β-actin is used as a loading control. **G** ATP level in the hippocampus (*n* = 4). **H-J** Representative Western blot and densitometric analysis of PSD95 and SYP levels in the hippocampal tissue lysates (*n* = 6). β-actin is used as a loading control. **K**, **M** Total arm entries (left) and spontaneous alteration ratio (right) in the Y-maze test (*n* = 10). **L**, **N-P** The Morris water maze (MWM) analysis is quantified to obtain the (**L**) swimming speed, (**N**) latency, (**O**) crossing times, and (**P**) target-zone duration (*n* = 10). **Q** Representative traces from the MWM test. The results are represented as mean ± SD. ^*^P < 0.05 versus Control + vehicle, ^#^*P* < 0.05 versus T2DM + vehicle group, ^**^P or ^##^*P* < 0.01, ^***^P or ^###^*P* < 0.001, ^****^P or.^####^*P* < 0.0001

To assess the functional relevance of mitophagy in neuronal function and memory ability, we also further quantified complex I-V expressions and ATP level. T2DM significantly decreased complex I-V expressions as well as ATP level, while these effects were ameliorated by UA (Fig. 4A-G). Additionally, UA not only re-upregulated PSD95 expression (Fig. 4H-J), but also significantly improved impaired hippocampus-related learning/memory in T2DM mice (Fig. 4K-Q). Similar to the Mdivi-1-treated mice, there was no effect on physiological and metabolic parameters of T2DM mice post UA administration (Fig S2 E–H). These findings demonstrated that the involvement of mitophagy in the mitochondrial and neuronal function and impairments in hippocampus-dependent memory induced by T2DM.

### Cav-1 overexpression in the neurons inhibits mitochondrial fission in T2DM mice via GSK3β/Drp1 pathway

**Fig. 5:**
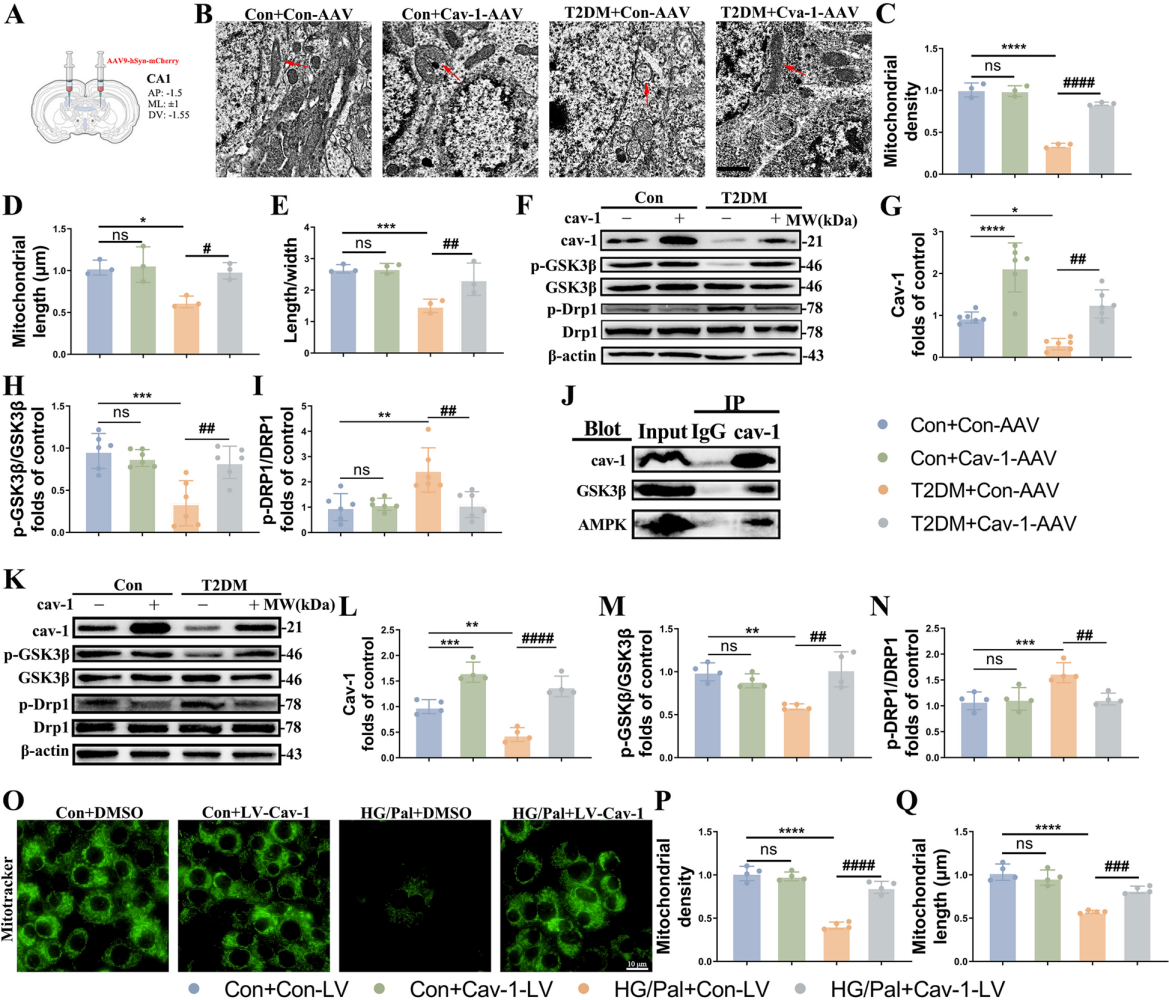
Cav-1 overexpression inhibits mitochondrial fission by GSK3β/Drp1 pathway. **A** Schematic figure of virus injection. The AAV vector was injected into left and right CA1 areas in the T2DM mouse. **B-E** Transmission electron microscopy showing mitochondrial morphology of neurons in the hippocampus of different groups (**B**), and measurements of mitochondrial density (**C**), length (**D**), ratio of length to width (**E**) (*n* = 3). Scale bar: 1 µm. **F-I** Representative Western blot and densitometric analysis of cav-1, p-Drp1/Drp1 and Drp1 in the hippocampal tissue lysates (*n* = 6). β-actin is used as a loading control. **J** Densitometry of immunoreactive bands (*n* = 3). **K-N** Representative Western blot and densitometric analysis of cav-1, p-Drp1/Drp1 and Drp1 in the HT22 cells of different groups (*n* = 4). β-actin is used as a loading control. **O-Q** Representative Mito-Tracker and densitometric analysis of mitochondrial density and length in HT22 cells from the different groups (*n* = 4). Scale bar: 10 µm. The results are represented as mean ± SD. ^*^P < 0.05 versus Control + Con-AAV/LV, ^#^*P* < 0.05 versus T2DM + Con-AAV/LV group, ^**^P or ^##^*P* < 0.01, ^***^P or ^###^*P* < 0.001, ^****^P or.^####^*P* < 0.0001

We and others have shown previously that cav-1 plays a protective role in T2DM induced DACD, and our results above demonstrated that T2DM increases mitochondrial fission and inhibits mitophagy to induce mitochondrial imbalance of hippocampal neurons. In view of this, we asked whether cav-1 protected hippocampal neurons from T2DM by regulating mitochondrial fission-mitophagy axis. We injected the AAVs carrying hSyn-promoter and cav-1 cDNA into the CA1 region to establish a mouse model overexpressing cav-1 (Fig. 5A). Unsurprisingly, TEM images and western blotting analysis indicated the T2DM mice with overexpressed cav-1 had preserved mitochondrial length and ultrastructure order (Fig. 5B-E), as well as a lower ratio of p-Drp1/Drp1 (Fig. 5F, G, I). Accordingly, HT22 cells transfected with cav1 lentivirus (LV) presented reduced phosphorylation of Drp1 (Fig. 5K, L, N), longer mitochondria and higher mitochondrial density (Fig. 5O-Q).

GSK3β has been reported to be activated in db/db mice owing to dephosphorylation on Ser9 residue and can mediate mitochondrial defects by modulating Drp1 Ser616 phosphorylation. Thus, we speculated that GSK3β/Drp1 signaling pathway could be the mechanism whereby cav-1 prevents T2DM induced excessive mitochondrial fission. In fact, results of the western blotting assay (Fig. 5F, I, K, M) and co-immunoprecipitation (Co-IP) confirmed cav-1 interacts with GSK3β and inactivates GSK3β by phosphorylation on Ser9 residue in a T2DM mouse model (Fig. 5J). These findings revealed that cav-1 upregulation protected neurons against T2DM-induced increased mitochondrial fission via GSK3β/Drp1 pathway.

### Neuron-targeted cav-1 overexpression enhanced mitophagy in T2DM mice via AMPK

**Fig. 6:**
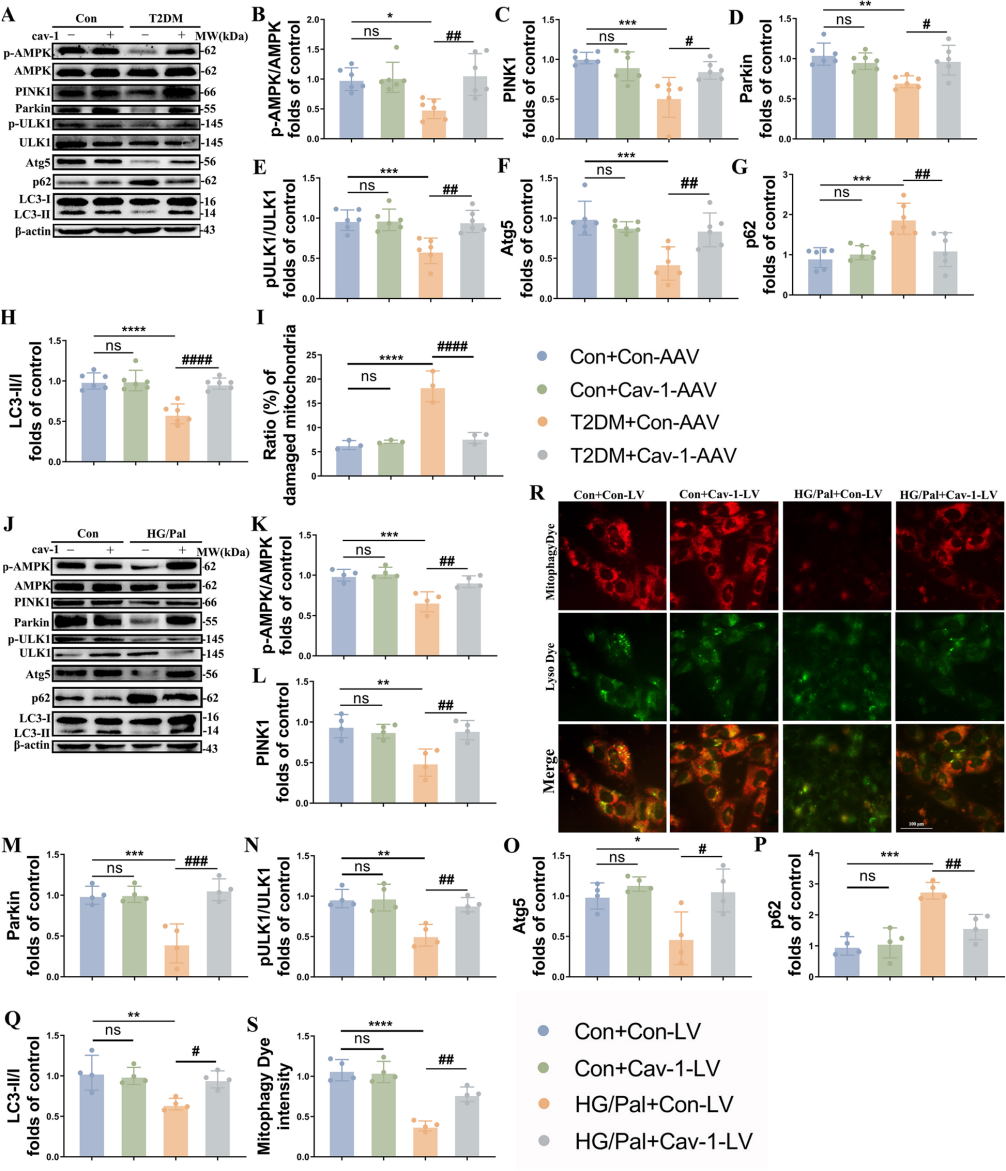
Cav-1 overexpression boosts mitophagy via AMPK pathway. **A-H** Representative Western blot and densitometric analysis of p-AMPK/AMPK, PINK1, Parkin, p-ULK1/ULK1, Atg5, p62 and ratio of LC3II/I in the hippocampal tissue lysates (*n* = 6). β-actin is used as a loading control. **I** The proportion of damaged mitochondria in the neurons of hippocampus (*n* = 3). **J-Q** Representative Western blot and densitometric analysis of p-AMPK/AMPK, PINK1, Parkin, p-ULK1/ULK1, Atg5, p62 and ratio of LC3II/I in the HT22 cells (*n* = 4). β-actin is used as a loading control. **R**, **S** Representative mitophagy dye and lyso dye and densitometric analysis of mitophagy dye intensity (*n* = 4). Scale bar: 100 µm. The results are represented as mean ± SD. ^*^P < 0.05 versus Control + Con-AAV/LV, ^#^*P* < 0.05 versus T2DM + Con-AAV/LV group, ^**^P or ^##^*P* < 0.01, ^***^P or ^###^*P* < 0.001, ^****^P or.^####^*P* < 0.0001

We went on to further explore the possibility that cav-1 prompts mitophagy during T2DM. As expected, we found that exogenous cav1 markedly upregulated the expressions of PINK1, Parkin, Atg5, p-ULK1/ULK1 and LC3II/I, whereas downregulated the expression of p62 in the T2DM mice (Fig. 6A, C-H). Furthermore, neuron-targeted cav-1 overexpression effectively rescued mitochondrial matrix density and organized cristae morphology, accompanied with decreased ratio of damaged mitochondria (Fig. 6I). In parallel to the results in vivo, cav-1-LV treatment activated the PINK1/Parkin and ULK1-dependent pathways (Fig. 6J, L-Q) and boosted mitochondria-lysosome interactions (Fig. 6R, S).

Based on previous studies and combined with the data we have obtained, we hypothesized that cav-1 might enhance mitophagy by increased phosphorylation of AMPK, which was verified by the following results. T2DM significantly inhibited AMPK phosphorylation at tyrosine 172 in the hippocampus, which were reversed by cav-1 overexpression (Fig. 6A, B, J, K. More than that, immunoprecipitation of cav-1 followed by immunoblotting with AMPK showed an AMPK immunoreactive band, which means cav-1 could directly regulate AMPK in T2DM-affected brain (Fig. 5J). These results demonstrated that cav-1-AMPK interaction mediates mitophagy under T2DM condition.

### Cav-1 upregulation ameliorated DACD by improving mitochondrial and neuronal functions

On the basis of the above findings, we hypothesized that cav-1 upregulation ameliorates DACD by maintaining mitochondrial homeostasis and neuronal function. To this end, we assessed the mitochondrial morphology and structure in hippocampal neurons from T2DM mice. The TEM results indicated T2DM augmented mitochondrial fission and increased damaged mitochondria, and the morphology of mitochondria was recapitulated in T2DM + Cav-1-AAV group (Fig. 5B). We also performed western blotting and ATP assay on hippocampal tissues, T2DM downregulated complex I-V expressions and ATP level, these effects were reversed upon cav-1 overexpression (Fig S4 A-G). Similarly, Cav-1-LV effectively increased the MMP of HG/Pal treated HT22 cells in vitro (Fig S4 H, I).

**Fig. 7:**
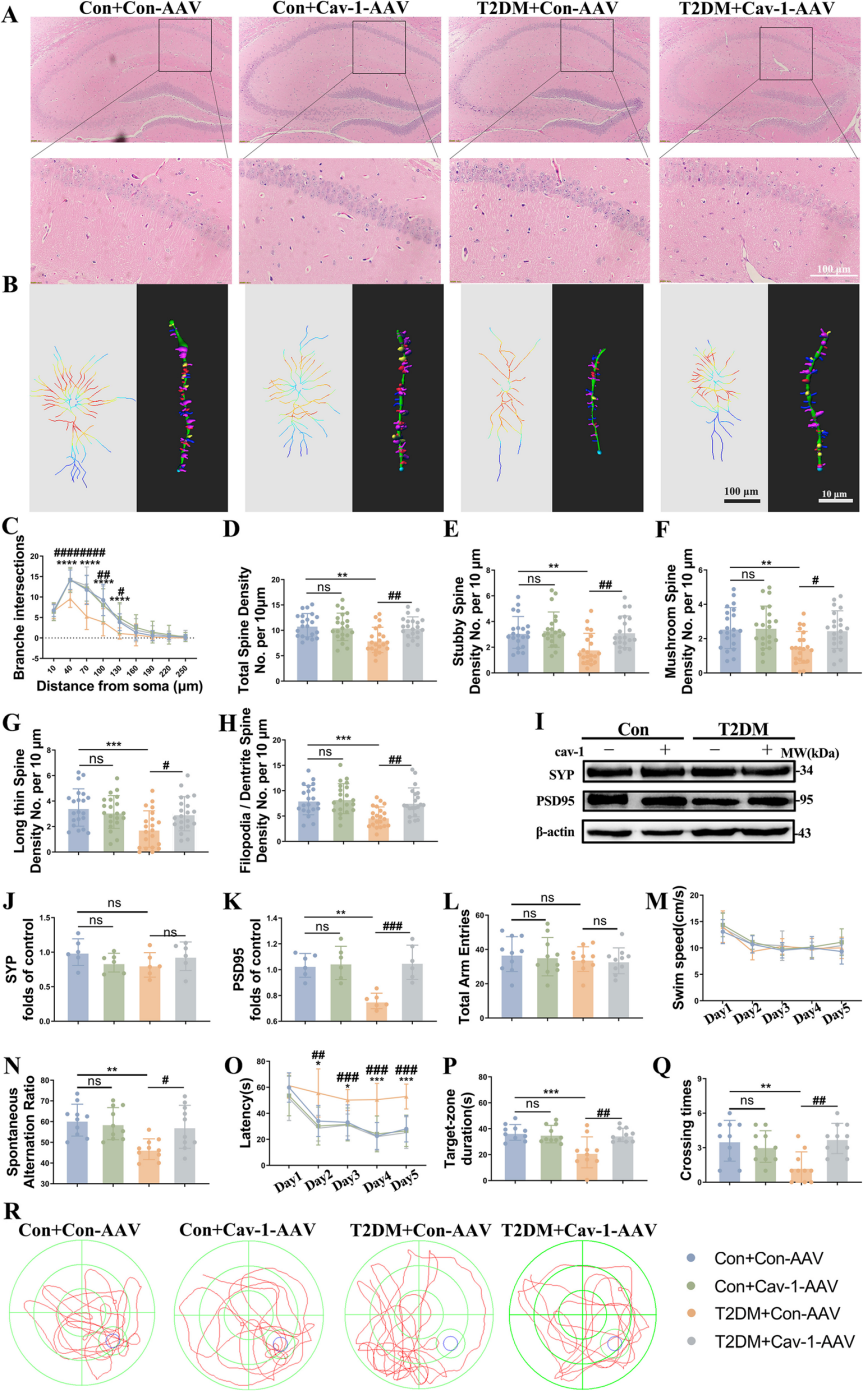
Cav-1 overexpression improves neuronal function and synaptic morphology, as well as cognitive ability in T2DM mice. **A** Representative HE staining of mouse hippocampus sections in each group. Scale bar: 100 µm. **B** Golgi imaging for the neuronal morphology (left) and 3D reconstruction of dendrites (right) in hippocampal pyramidal neurons of CA1 region in the experimental groups (*n* = 3). Scale bar: 100 (left) and 10 (right) µm. **C** Statistical analysis for the dendritic complexity. (**D-G**) Quantification of the total spine density (**D**), stubby spine density (**E**), mushroom spine density (**F**), long thin spine density (**G**), and filopodia/dendrite spine density (**H**) in hippocampal CA1 pyramidal neurons of each group. **I-K** Representative Western blot and densitometric analysis of PSD95 and SYP levels in the hippocampal tissue lysates (*n* = 6). β-actin is used as a loading control. **L**, **N** Total arm entries (left) and spontaneous alteration ratio (right) in the Y-maze test (*n* = 10). **M**, **O-Q** The Morris water maze (MWM) analysis is quantified to obtain the (**M**) swimming speed, (**O**) latency, (**P**) crossing times, and (**Q**) target-zone duration (*n* = 10). **R** Representative traces from the MWM test. The results are represented as mean ± SD. ^*^P < 0.05 versus Control + Con-AAV, ^#^*P* < 0.05 versus T2DM + Con-AAV group, ^**^P or ^##^*P* < 0.01, ^***^P or ^###^*P* < 0.001, ^****^P or.^####^*P* < 0.0001

Consistent with our previous study, HE staining results demonstrated that the neurons in the hippocampal CA1 region of T2DM mice exhibited high ratio of nuclear-cytoplasmic, vacuolated cytoplasm, and hyperchromatic nuclei, while the neurons of the control group were regularly arranged with distinct cytoplasmic borders (Fig. 7A). We additionally performed Golgi staining to reconstructed hippocampal CA1 pyramidal cells for morphometric analysis (Fig. 7B). T2DM + cav-1-AAV mice produced significant increases in the number of branch points of regions located 40 mm, 70 mm, 100 mm, and 130 mm from the center of soma, but T2DM + con-AAV mice did not **(**Fig. 7C**)**. The complexity of neuronal processes was reduced in T2DM + con-AAV group, as manifested by lower numbers of total spine density and various neuronal spine densities, while these structural defects were reversed by cav-1 overexpression (Fig. 7D-G). Also, cav-1 upregulation markedly increased PSD95 expression (Fig. 7H-J).

In parallel to the previous results, re-expression of cav-1 improved cognitive performance in the T2DM mice (Fig. 7K-Q). The data up to this point suggested that the restoration of cav-1 facilitated cognitive function by protecting hippocampal neuronal morphology and function in the T2DM condition.

## Discussion

In the present study, our data showed that (1) T2DM induced excessive mitochondrial fission and suppressed mitophagy in the hippocampus; (2) cav-1 overexpression conferred substantial protective effects against DACD by preserving mitochondrial fission-mitophagy axis; (3) GSK3β/Drp1 signaling served as a novel down-stream target of mitochondrial fission inhibition by cav-1; (4) the interaction between cav-1 and AMPK is the key for cav-1 to promote mitophagy during T2DM.

DACD is a disease state in which DM leads to cognitive decline and dementia, especially T2DM, is closely associated with a high rate of mortality [36, 37]. In spite of the pathogenesis of DACD has been described in lots of in-depth studies, precise pathophysiological mechanisms for DACD have not yet been elucidated, making it somewhat challenging to engage effective therapeutic intervention for DACD. Understanding molecular mechanisms in the hippocampus of T2DM mice model will likely generate novel approaches for the treatment of T2DM-induced DACD.

There is already a large evidence base to suggest that mitochondria, the "gatekeeper" for functional bioenergetics and balanced redox homeostasis, represent an emerging drug target for many age-associated neurological disorders, including T2DM-induced DACD [38–40]. Consistent with our previous results [39], we found that under T2DM condition, the redox homeostasis was destructed, which impair mitochondrial respiration function, leading to neuronal energy disorder. Additionally, rescued mitochondrial activity within compromised neurons could effectively improve neuronal function and recover cognitive ability in T2DM mice.

Mitochondria are cytoplasmic organelles that undergo morphologic changes for the adaption to cellular energetic demands. These changes can occur through the flux of iterative fusion and fission events, which are termed as mitochondrial dynamics [41]. When faced with excessive cellular stress, mitochondrial fission procedures are responsible for separating pre-degraded mitochondria that was damaged [42, 43], whereas the complementation between functional mitochondria and dysfunctional mitochondria, enabling survival of dysfunctional mitochondria, was facilitated through fusion [44]. Mitochondrial fission precedes neuronal death after opposing T2DM condition [15, 17, 45], which is mediated by Drp1 (a cytosolic protein that initiates mitochondrial fission through its recruitment to the mitochondrial surface, which contributes to the phosphorylation at serine616) pathway [18]. Consistently, our data suggested T2DM shifted the balance of mitochondrial fission and fusion towards fission in the hippocampus of mice, neuronal mitochondria began to swell and fragment into small individual mitochondria, resulted by up-regulation of pDrp1 expression. While mitochondrial fission inhibitor Mdivi-1 treatment showed an opposite outcome both in vitro and in vivo. In addition, Mdivi-1 significantly rescued T2DM-induced DACD by improving mitochondrial and neuronal function.

In the mitochondrial life cycle, fission events enable both biogenesis of a healthy pool of newly synthesized mitochondria and clearance of damaged mitochondria via mitophagy, a special form of autophagy and a protective mechanism that selectively degrades dysfunctional mitochondria to sustain mitochondrial function and structure following damage or stress [46]. Ample evidence points towards that T2DM may lead to accumulated mitochondrial DNA mutations from oxidative stress, which induces mitophagy-resistant mitochondria to aggregate, resulting in mitophagy arrest [47, 48]. Such defects in mitophagy have been reported to cause decreased synaptic function due to stress-induced glucocorticoids [49]. Combining the above evidence can raise a reasonable hypothesis that T2DM causes excessive fission and defective mitophagy, which dramatically mediated mitochondrial dysfunction. Previous researches have proved ULK1 protein and PINK1/Parkin pathway are both critical to promote mitophagy [33, 50]. ULK1, for its part, is determined as a critical element of the mitophagic integrated system. In some cases, it may additionally connect with PINK1/Parkin-mediated mitophagy [51]. More than that, ULK1 can also directly phosphorylate mitophagy receptors such as FUNDC1, BNIP3, NIX and so on [50, 52, 53]. It is worth noting that, impairment in the PINK1/Parkin and ULK1-dependent pathways have been observed in both T2DM and neurodegeneration [33, 54, 55].

In accordance with these previous findings, our in vivo and in vitro analyses revealed for the first time that long-term T2DM environment could serve as a driving factor to induce an imbalance in mitochondrial fission and mitophagy, which in turn led to mitochondrial dysfunction and then neuronal function impairment, leading to DACD. Herein, we found T2DM mice hippocampus developed lower expressions of PINK1, Parkin, Atg5 and LC3II/I, downregulated phosphorylation of ULK1, elevated p62 expression, swollen mitochondria with irregular cristae morphology, accompanied by a cascade of events such as mitochondrial dysfunction, neuronal damage and cognitive impairment, which were reversed by UA treatment. Interestingly, physiological and metabolic parameters were not affected by either Mdivi-1 or UA, which means that the anti-T2DM protective effects of them were actually due to the improved mitochondrial quality control, instead of improving diabetes-associated metabolic defects.

It is extensively recognized that cav-1 is closely associated with the pathophysiological processes and risk factors of neurodegenerative diseases [24]. In terms of DACD, we have previously shown that cav-1 attenuates DACD by modulating neuronal ferroptosis-mediated mitochondrial homeostasis. Taking into account the effects of cav-1 in DACD and based on the findings discussed above, here we speculated cav-1 can regulate mitochondrial fission-mitophagy axis to control mitochondrial quality. The following results from our study supported this idea. Specific overexpression of cav-1-targeted hippocampal neurons exerted a protective action on imbalanced mitochondrial dynamics of T2DM mice. Up-regulation of cav-1 not only effectively inhibited increased mitochondrial fission, but also significantly activated defective mitophagy in the T2DM group. Meanwhile, mitochondrial morphology and function, neuronal plasticity and cognitive ability were improved by overexpressing cav-1.

It is rational to seek therapeutic strategies to rebalance the disrupted mitochondrial fission-mitophagy axis to preserve mitochondrial function. For this reason, we further investigated the specific mechanism by which cav-1 modulates mitochondrial fission-mitophagy axis. GSK3β has already been pointed out to be activated in either T2DM-affected hippocampus or high glucose-treated human SK cell lines owing to dephosphorylation at serine 9, which is essential to prevent Drp1 phosphorylation on Ser616 under T2DM condition in vitro and in vivo [15, 56, 57]. And blockage of GSK3β-regulated Drp1 phosphorylation could provide neuroprotection in neuronal and mouse models of Alzheimer's disease [58]. Here, coincided with previous studies, in our T2DM mouse model, we found the level of p-GSK3β Ser9 was significantly decreased compared with the control group, the similar trend was observed in HT22 cells. However, cav-1 overexpression efficiently inactivated GSK3β by phosphorylating GSK3β in vitro and in vivo. We then further demonstrated cav-1 can interact with GSK3β by Co-IP in vivo. Hence, we believe the interaction between cav-1 and GSK3β may lie, at least in part, in the blockage of excessive mitochondrial fission.

On the other hand, AMPK, an energy sensor with aberrant expression in multiple diseases, appears to have a major impact on the development of DM and related complications [59]. In recent years, AMPK has received an increasing amount of attention as a key regulatory factor of mitochondrial metabolism and mitophagy in metabolic disorders [60, 61]. In fact, it has been reported that AMPK implicates in mitochondrial fission and possibly interplays with PINK1/Parkin signaling [54]. Mitophagy can reportedly be modulated through PINK1/Parkin pathway, which in turn is positively regulated by AMPK via phosphorylation on Thr172 [31, 62–64]. AMPK also mediated ULK1 phosphorylation at Ser555 to regulate mitophagy in various cell types [29, 54, 65]. In our present study, we observed marked reduced p-AMPK Thr172 along with much lower expression levels of PINK1, Parkin and p-ULK1 in T2DM condition both in vivo and in vitro, while these changes were reversed by cav-1. Similarly, we also confirmed the interaction between cav-1 and AMPK by Co-IP in vivo. These results demonstrated that cav-1 rescued mitophagy primarily by interacting with AMPK to activate PINK1/Parkin and ULK1-dependent pathways under T2DM condition.

In summary, we demonstrated that cav-1 improved T2DM-induced DACD by blocking excessive mitochondrial fission and stimulating defective mitophagy, respectively. This is the first evidence of an imbalance between mitochondrial fission and mitophagy in a long-term T2DM environment, which provides new insights into our understanding the role of mitochondrial dynamic equilibrium in the pathogenesis of DACD, highlighting the opportunity of a novel therapy for DACD.

## Material and methods

### Animals

As described in our previous study [26], male C57BL/6 J mice, aged 4 weeks (weighting 14–17 g), were purchased from the Laboratory Animal Center of Xi’an Jiaotong University (Xi’an, Shanxi, China, 2018–107). Mice were housed in a 12 h light/dark cycle with food and tap water ad libitum. All animal experimental protocols were approved by Xi’an Jiaotong University institutional Animal Care and conducted in accordance with the ARRIVE guidelines.

T2DM model was conducted following a method described previously [26]. Schematic model of this study is shown in Supplementary Figure S1.

### In vivo drug treatment with Mdivi-1 and Urolithin A (UA)

Mice were injected intraperitoneally with either vehicle, Mdivi-1 (25 mg/kg body weight, Selleck, USA) and UA (2.5 mg/kg body weight, Selleck, USA) daily in week 23. The Mdivi-1 and UA treatment lasted two and eight weeks respectively. Mdivi-1 and UA were dissolved in vehicle (2%DMSO + 50%PEG300 + 5%Tween80 + ddH_2_O).

### Stereotaxic injection

Stereotaxic injection was performed as reported previously [26]. The Supplemental Methods provide the detailed protocols.

### HT22 cell culture and treatment

Immortalized mouse HT22 hippocampal neurons (Beina Chuanglian Biotech Institute, Beijing, China) were cultured in Dulbecco's modified Eagle's medium (DMEM, SH30022.01, HyClone, USA); supplemented with 10% fetal bovine serum (Gibco Life Technologies, USA), 1% penicillin; cultured at 37 °C in 5% CO_2_. The DMEM was changed every three days.

As previously described [26], high glucose (HG) plus palmitate (Pal) was used in vitro experiments to mimic HFD and STZ-induced in vivo model of DM. Mdivi-1 (1 μM, Selleck, USA)/UA (100 μM, MCE, USA)/vehicle was administered with HG/Pal treatment on HT22 cells for 24 h.

### Co-immunoprecipitation (Co-IP)

The Co-IP experiments performed with the Pierce Co-IP Kit (Thermo Fisher Scientific, Waltham, MA, USA) following the manufacturer’s protocol. Immune complexes collected from hippocampal tissue lysate were detected by western blotting.

### Western blotting

The hippocampal samples from mice and the HT22 cellular samples were prepared and subjected to western blotting analysis as described previously [26, 65]. The Supplemental Methods provide the detailed protocols. List of antibodies involved in the western blotting is shown in Supplementary Table S1.

### Behavioral tests

Y maze and Morris water maze (MWM) tests were performed in week 28 as described earlier in detail [26]. The Supplemental Methods provide the detailed protocols.

### Hematoxylin–eosin (HE) staining

HE staining was performed as described in detail previously by us [26]. The Supplemental Methods provide the detailed protocols.

### Mito-Tracker staining

Mitochondria in HT22 cells were visualized with the mitochondria-sensitive dye Mito-Tracker Green (Beyotime Biotechnology, China). Cells plated on small dishes were cultured to 70% density. After different treatments, cells were incubated in Mito-Tracker Green staining solution (0.2 μM) at 37 °C for 1 h. Subsequently, the small dishes were washed with PBS, fluorescent images of live cells were detected by 490/516 nm using an inverted fluorescence microscope (Olympus).

### Mitophagy detection

For the detection of mitophagy in HT22 cells, a novel Mitophagy Detection Kit (Dojindo Molecular Technologies, Rockville, USA) was used. Cells were washed twice with Hanks’ balanced salt buffer and afterwards incubated at 37 °C for 1 h with 100 nmol Mtphagy Dye diluted in DMEM. Subsequently, discard the supernatant and wash the cells with Hanks’ balanced salt buffer. After different treatments, cells were incubated at 37 °C for 1 h with 1 μmol/l Lyso Dye to observe the co-localization of Mtphagy Dye and lysosome. After washing with Hanks’ balanced salt buffer, the cells were scanned with an inverted fluorescence microscope (Olympus).

### JC-1 staining

JC-1 staining was performed as previously described [26]. The Supplemental Methods provide the detailed protocols.

### ATP detection

ATP level was measured using the specific kit in accordance with the instructions (Beyotime Biotechnology, Shanghai, CN, S0027).

### Serum insulin measurement

Serum insulin was measured as described in detail previously by us and Liu et al. [26, 66]. The Supplemental Methods provide the detailed protocols.

### Golgi staining

Golgi staining was performed as described in detail previously by us and Long et al. [26, 67]. The Supplemental Methods provide the detailed protocols.

### Transmission electron microscope (TEM)

The experimental procedure for TEM was performed as described in detail previously by us and Stoica et al. [26, 68]. The Supplemental Methods provide the detailed protocols.

### Statistical analysis

All data results were expressed as the mean ± standard error of the mean (S.E.M.). All data were analyzed with GraphPad Prism software (version 9.0). Two-way repeated measures ANOVA was applied for analyzing the MWM (group × day). Comparisons between groups were performed with one-way ANOVA followed by Tukey or Dunnett test. Statistical significance was set at *P* < 0.05.

### Supplementary Information

**Additional file 1.** Materials and Methods.

**Additional file 2:** **Figure S1.** The general procedure of this study in vivo.

**Additional file 3:** **Figure S2.** Mdivi-1 and UA did not exert effects by altering the physiological metabolism.

**Additional file 4: Figure S3.** Mitophagy is decreased in the HG/Pal-treated HT22 cells.

**Additional file 5: Figure S4.** Cav-1 overexpression recovers mitochondrial function in T2DM environment.

**Additional file 6: Figure S5.**

**Additional file 7:** **Table S1.** The list of primary antibodies.

## Data Availability

Not applicable.

## Abbreviations

DM: Diabetes mellitus
T2DM: Type 2 diabetes mellitus
STZ: Streptozotocin
HFD: High fat diet
DACD: Diabetes-associated cognitive dysfunction
Drp1: Dynamin-related protein-1
GSK3β: Glycogen synthase kinase 3β
PINK1: PTEN-induced kinase 1
TEM: Transmission electron microscopy
Atg5: Autophagy protein 5
cav-1: Caveolin-1
AMPK: AMP-activated protein kinase
ULK1: Atg1/Unc-51 like autophagy activating kinase 1
OXPHOS: Oxidative phosphorylation
SYP: Synaptophysin
MWM: Morris water maze
HG/Pal: High glucose/palmitic acid
CNS: Central nervous system
MMP: Mitochondrial membrane potential
HOMA-IR: Homeostasis model assessment of insulin resistance

## References

[1] Livingston G, Huntley J, Sommerlad A, Ames D, Ballard C, Banerjee S (2020). Dementia prevention, intervention, and care: 2020 report of the Lancet Commission. The Lancet.

[2] Biessels GJ, Despa F (2018). Cognitive decline and dementia in diabetes mellitus: mechanisms and clinical implications. Nat Rev Endocrinol.

[3] Rawlings AM, Sharrett AR, Albert MS, Coresh J, Windham BG, Power MC (2019). The Association of Late-Life Diabetes Status and Hyperglycemia With Incident Mild Cognitive Impairment and Dementia: The ARIC Study. Diabetes Care.

[4] Dove A, Shang Y, Xu W, Grande G, Laukka EJ, Fratiglioni L (2021). The impact of diabetes on cognitive impairment and its progression to dementia. Alzheimers Dement.

[5] van Gennip ACE, Stehouwer CDA, van Boxtel MPJ, Verhey FRJ, Koster A, Kroon AA (2021). Association of Type 2 Diabetes, According to the Number of Risk Factors Within Target Range, With Structural Brain Abnormalities, Cognitive Performance, and Risk of Dementia. Diabetes Care.

[6] Chohan H, Senkevich K, Patel R, Bestwick J, Jacobs B, Bandres Ciga S (2021). Type 2 Diabetes as a Determinant of Parkinson's Disease Risk and Progression. Movement disorders : official journal of the Movement Disorder Society.

[7] International Diabetes Federation I. IDF Diabetes Atlas, 10th ed. 2021.

[8] Deshwal S, Fiedler K, Langer T (2020). Mitochondrial Proteases: Multifaceted Regulators of Mitochondrial Plasticity. Annu Rev Biochem.

[9] Cheng X, Huang N, Sheng Z (2022). Programming axonal mitochondrial maintenance and bioenergetics in neurodegeneration and regeneration. Neuron.

[10] Sabouny R, Shutt TE (2020). Reciprocal Regulation of Mitochondrial Fission and Fusion. Trends Biochem Sci.

[11] Rovira-Llopis S, Bañuls C, Diaz-Morales N, Hernandez-Mijares A, Rocha M, Victor VM (2017). Mitochondrial dynamics in type 2 diabetes: Pathophysiological implications. Redox Biol.

[12] Youle RJ, Narendra DP (2011). Mechanisms of mitophagy. Nat Rev Mol Cell Biol.

[13] Sun D, Wang J, Toan S, Muid D, Li R, Chang X (2022). Molecular mechanisms of coronary microvascular endothelial dysfunction in diabetes mellitus: focus on mitochondrial quality surveillance. Angiogenesis.

[14] Yu L, Dong X, Xue X, Xu S, Zhang X, Xu Y (2021). Melatonin attenuates diabetic cardiomyopathy and reduces myocardial vulnerability to ischemia-reperfusion injury by improving mitochondrial quality control: Role of SIRT6. J Pineal Res.

[15] Huang S, Wang Y, Gan X, Fang D, Zhong C, Wu L (2015). Drp1-mediated mitochondrial abnormalities link to synaptic injury in diabetes model. Diabetes.

[16] He C, Gao P, Cui Y, Li Q, Li Y, Lu Z et al. Low-glucose-sensitive TRPC6 dysfunction drives hypoglycemia-induced cognitive impairment in diabetes. (2001–1326 (Print)). 10.1002/ctm2.205 PMC7568851 33135341

[17] Maneechote C, Chunchai T, Apaijai N, Chattipakorn N, Chattipakorn S (2022). Pharmacological Targeting of Mitochondrial Fission and Fusion Alleviates Cognitive Impairment and Brain Pathologies in Pre-diabetic Rats. Mol Neurobiol.

[18] Fonseca TB, Sánchez-Guerrero Á, Milosevic I, Raimundo N (2019). Mitochondrial fission requires DRP1 but not dynamins. Nature.

[19] Kashatus DF, Lim K-H, Brady DC, Pershing NLK, Cox AD, Counter CM (2011). RALA and RALBP1 regulate mitochondrial fission at mitosis. Nat Cell Biol.

[20] Fivenson EM, Lautrup S, Sun N, Scheibye-Knudsen M, Stevnsner T, Nilsen H (2017). Mitophagy in neurodegeneration and aging. Neurochem Int.

[21] Xian H, Liou Y-C (2021). Functions of outer mitochondrial membrane proteins: mediating the crosstalk between mitochondrial dynamics and mitophagy. Cell Death Differ.

[22] Paul S, Saha D, Bk B (2021). Mitochondrial Dysfunction and Mitophagy Closely Cooperate in Neurological Deficits Associated with Alzheimer's Disease and Type 2 Diabetes. Mol Neurobiol.

[23] Su C-J, Shen Z, Cui R-X, Huang Y, Xu D-L, Zhao F-L (2020). Thioredoxin-Interacting Protein (TXNIP) Regulates Parkin/PINK1-mediated Mitophagy in Dopaminergic Neurons Under High-glucose Conditions: Implications for Molecular Links Between Parkinson’s Disease and Diabetes. Neurosci Bull.

[24] Tang W, Li Y, Li Y, Wang Q (2021). Caveolin-1, a Novel Player in Cognitive Decline. Neurosci Biobehav Rev.

[25] Cohen AW, Hnasko R, Schubert W, Lisanti MP (2004). Role of caveolae and caveolins in health and disease. Physiol Rev.

[26] Tang WXLY, He SX, Jiang T, Wang N, Du MY (2022). Caveolin-1 Alleviates Diabetes-Associated Cognitive Dysfunction Through Modulating Neuronal Ferroptosis-Mediated Mitochondrial Homeostasis. Antioxid Redox Signal.

[27] Tang WX, Li YS, He SX, Jiang T, Wang N, Du MY et al. Caveolin-1 Alleviates Diabetes-Associated Cognitive Dysfunction Through Modulating Neuronal Ferroptosis-Mediated Mitochondrial Homeostasis. Antioxidants & Redox Signaling.20. doi:10.1089/ars.2021.0233. 10.1089/ars.2021.0233 35350885

[28] Cai C, Guo Z, Chang X, Li Z, Wu F, He J (2022). Empagliflozin attenuates cardiac microvascular ischemia/reperfusion through activating the AMPKα1/ULK1/FUNDC1/mitophagy pathway. Redox Biol.

[29] Laker RC, Drake JC, Wilson RJ, Lira VA, Lewellen BM, Ryall KA (2017). Ampk phosphorylation of Ulk1 is required for targeting of mitochondria to lysosomes in exercise-induced mitophagy. Nat Commun.

[30] Han Y, Tang S, Liu Y, Li A, Zhan M, Yang M (2021). AMPK agonist alleviate renal tubulointerstitial fibrosis via activating mitophagy in high fat and streptozotocin induced diabetic mice. Cell Death Dis.

[31] Cao S, Xiao H, Li X, Zhu J, Gao J, Wang L et al. AMPK-PINK1/Parkin Mediated Mitophagy Is Necessary for Alleviating Oxidative Stress-Induced Intestinal Epithelial Barrier Damage and Mitochondrial Energy Metabolism Dysfunction in IPEC-J2. Antioxidants (Basel, Switzerland). 2021;10(12). doi:10.3390/antiox10122010. 10.3390/antiox10122010 PMC8698696 34943113

[32] Dong Y, Yu M, Wu Y, Xia T, Wang L, Song K et al. Hydroxytyrosol Promotes the Mitochondrial Function through Activating Mitophagy. Antioxidants (Basel, Switzerland). 2022;11(5). doi:10.3390/antiox11050893. 10.3390/antiox11050893 PMC9138034 35624756

[33] Kerr JS, Adriaanse BA, Greig NH, Mattson MP, Cader MZ, Bohr VA (2017). Mitophagy and Alzheimer’s Disease: Cellular and Molecular Mechanisms. Trends Neurosci.

[34] Lin J, Zhuge J, Zheng X, Wu Y, Zhang Z, Xu T (2020). Urolithin A-induced mitophagy suppresses apoptosis and attenuates intervertebral disc degeneration via the AMPK signaling pathway. Free Radical Biol Med.

[35] Zhang Y, Aisker G, Dong H, Halemahebai G, Zhang Y, Tian L (2021). Urolithin A suppresses glucolipotoxicity-induced ER stress and TXNIP/NLRP3/IL-1β inflammation signal in pancreatic β cells by regulating AMPK and autophagy. Phytomedicine.

[36] Shudan, Gao, Yaojing, Chen, Feng, Sang et al. White Matter Microstructural Change Contributes to Worse Cognitive Function in Patients With Type 2 Diabetes. Diabetes. 2019;68(11):2085–94. 10.2337/db19-0233 PMC6804632 31439643

[37] Sloten T, Sedaghat S, Carnethon MR, Launer LJ, Stehouwer C. Cerebral microvascular complications of type 2 diabetes: stroke, cognitive dysfunction, and depression. The Lancet Diabetes & Endocrinology. 2020;8(4). 10.1016/S2213-8587(19)30405-X PMC11044807 32135131

[38] Amorim J, Coppotelli G, Rolo A, Palmeira C, Ross J, Sinclair D (2022). Mitochondrial and metabolic dysfunction in ageing and age-related diseases. Nat Rev Endocrinol.

[39] Ma H, Jiang T, Tang W, Ma Z, Pu K, Xu F (2020). Transplantation of platelet-derived mitochondria alleviates cognitive impairment and mitochondrial dysfunction in db/db mice. Clin Sci.

[40] Li C, Chen C, Qin H, Ao C, Chen J, Tan J et al. The Role of Mitochondrial Dynamin in Stroke. (1942–0994 (Electronic)). 10.1155/2022/2504798 PMC9106451 35571256

[41] Kraus F, Roy K, Pucadyil T, Ryan M (2021). Function and regulation of the divisome for mitochondrial fission. Nature.

[42] Twig G, Hyde B, Shirihai OS. Mitochondrial fusion, fission and autophagy as a quality control axis: The bioenergetic view. Biochimica et Biophysica Acta (BBA) - Bioenergetics. 2008;1777(9):1092–7. doi:10.1016/j.bbabio.2008.05.001. 10.1016/j.bbabio.2008.05.001 PMC3809017 18519024

[43] Malena A, Loro E, Di Re M, Holt IJ, Vergani L (2009). Inhibition of mitochondrial fission favours mutant over wild-type mitochondrial DNA. Hum Mol Genet.

[44] Youle RJ, Bliek AMvd. Mitochondrial Fission, Fusion, and Stress. Science. 2012;337(6098):1062–5. doi:doi:10.1126/science.1219855. 10.1126/science.1219855 PMC4762028 22936770

[45] Hei C, Zhou Y, Zhang C, Gao F, Cao M, Yuan S (2022). Rapamycin ameliorates brain damage and maintains mitochondrial dynamic balance in diabetic rats subjected to middle cerebral artery occlusion. Metab Brain Dis.

[46] Burman J, Pickles S, Wang C, Sekine S, Vargas J, Zhang Z (2017). Mitochondrial fission facilitates the selective mitophagy of protein aggregates. J Cell Biol.

[47] Carvalho C, Machado N, Mota PC, Correia SC, Cardoso S, Santos RX (2013). Type 2 diabetic and Alzheimer's disease mice present similar behavioral, cognitive, and vascular anomalies. Journal of Alzheimer's disease : JAD.

[48] Bhansali S, Bhansali A, Walia R, Saikia UN, Dhawan V (2017). Alterations in Mitochondrial Oxidative Stress and Mitophagy in Subjects with Prediabetes and Type 2 Diabetes Mellitus. Front Endocrinol.

[49] Choi G, Lee H, Chae C, Cho J, Jung Y, Kim J (2021). BNIP3L/NIX-mediated mitophagy protects against glucocorticoid-induced synapse defects. Nat Commun.

[50] Wu W, Tian W, Hu Z, Chen G, Huang L, Li W (2014). ULK1 translocates to mitochondria and phosphorylates FUNDC1 to regulate mitophagy. EMBO Rep.

[51] Silvian L (2022). PINK1/Parkin Pathway Activation for Mitochondrial Quality Control - Which Is the Best Molecular Target for Therapy?. Frontiers in aging neuroscience.

[52] Poole LP, Bock-Hughes A, Berardi DE, Macleod KF (2021). ULK1 promotes mitophagy via phosphorylation and stabilization of BNIP3. Sci Rep.

[53] Murakawa T, Okamoto K, Omiya S, Taneike M, Yamaguchi O, Otsu K (2019). A Mammalian Mitophagy Receptor, Bcl2-L-13, Recruits the ULK1 Complex to Induce Mitophagy. Cell Rep.

[54] Iorio R, Celenza G, Petricca S. Mitophagy: Molecular Mechanisms, New Concepts on Parkin Activation and the Emerging Role of AMPK/ULK1 Axis. Cells. 2021;11(1). doi:10.3390/cells11010030. 10.3390/cells11010030 PMC8750607 35011593

[55] Franco-Iborra S, Plaza-Zabala A, Montpeyo M, Sebastian D, Vila M, Martinez-Vicente M (2021). Mutant HTT (huntingtin) impairs mitophagy in a cellular model of Huntington disease. Autophagy.

[56] Hu D, Sun X, Magpusao A, Fedorov Y, Thompson M, Wang B (2021). Small-molecule suppression of calpastatin degradation reduces neuropathology in models of Huntington's disease. Nat Commun.

[57] Liu J, Li L, Xie P, Zhao X, Shi D, Zhang Y (2022). Sevoflurane induced neurotoxicity in neonatal mice links to a GSK3β/Drp1-dependent mitochondrial fission and apoptosis. Free Radical Biol Med.

[58] Yan J, Liu X, Han M, Wang Y, Sun X, Yu N (2015). Blockage of GSK3β-mediated Drp1 phosphorylation provides neuroprotection in neuronal and mouse models of Alzheimer's disease. Neurobiol Aging.

[59] Entezari M, Hashemi D, Taheriazam A, Zabolian A, Mohammadi S, Fakhri F et al. AMPK signaling in diabetes mellitus, insulin resistance and diabetic complications: A pre-clinical and clinical investigation. Biomedicine & pharmacotherapy = Biomedecine & pharmacotherapie. 2022;146:112563. doi:10.1016/j.biopha.2021.112563. 10.1016/j.biopha.2021.112563 35062059

[60] Herzig S, Shaw RJ (2018). AMPK: guardian of metabolism and mitochondrial homeostasis. Nat Rev Mol Cell Biol.

[61] Liu L, Liao X, Wu H, Li Y, Zhu Y, Chen Q (2020). Mitophagy and Its Contribution to Metabolic and Aging-Associated Disorders. Antioxid Redox Signal.

[62] Chen C, Chen Y, Liu T, Song D, Ma D, Cheng O (2022). Dexmedetomidine Can Enhance PINK1/Parkin-Mediated Mitophagy in MPTP-Induced PD Mice Model by Activating AMPK. Oxid Med Cell Longev.

[63] Jin Z, Chang B, Wei Y, Yang Y, Zhang H, Liu J et al. Curcumin exerts chondroprotective effects against osteoarthritis by promoting AMPK/PINK1/Parkin-mediated mitophagy. Biomedicine & pharmacotherapy = Biomedecine & pharmacotherapie. 2022;151:113092. doi:10.1016/j.biopha.2022.113092. 10.1016/j.biopha.2022.113092 35550528

[64] de Marañón A, Díaz-Pozo P, Canet F, Díaz-Morales N, Abad-Jiménez Z, López-Domènech S (2022). Metformin modulates mitochondrial function and mitophagy in peripheral blood mononuclear cells from type 2 diabetic patients. Redox Biol.

[65] Jang JE, Eom J-I, Jeung H-K, Cheong J-W, Lee JY, Kim JS, et al. Targeting AMPK-ULK1-mediated autophagy for combating BET inhibitor resistance in acute myeloid leukemia stem cells. Autophagy. 2017;13(4):761–2. 10.1080/15548627.2016.1278328. 10.1080/15548627.2016.1278328 PMC5388226 28118076

[66] Liu Z, Dai X, Zhang H, Shi R, Hui Y, Jin X et al. Gut microbiota mediates intermittent-fasting alleviation of diabetes-induced cognitive impairment. Nat Commun. 2020;11(1):855. 10.1038/s41467-020-14676-4. 10.1038/s41467-020-14676-4 PMC7029019 32071312

[67] Luo Q, Xian P, Wang T, Wu S, Sun W, et al. Antioxidant activity of mesenchymal stem cell-derived extracellular vesicles restores hippocampal neurons following seizure damage. Theranostics. 2021;11(12):5986-6005. 10.7150/thno.58632. 10.7150/thno.58632 PMC8058724 33897894

[68] Makarevich O, Sabirzhanov B, Aubrecht T, Glaser E, Polster Henry R, et al. Mithramycin selectively attenuates DNA-damage-induced neuronal cell death. Cell Death Dis. 2020;11(7):587. 10.1038/s41419-020-02774-6. 10.1038/s41419-020-02774-6 PMC7385624 32719328

