# Supplementary material for: Neuron-targeted overexpression of caveolin-1 alleviates diabetes-associated cognitive dysfunction via regulating mitochondrial fission-mitophagy axis
Source: Cell Commun Signal. 2023 Dec 15;21:357. doi: 10.1186/s12964-023-01328-5 (PMC10722701; doi:10.1186/s12964-023-01328-5)
Supplement: Supplementary file 2 — Additional file 1. Materials and Methods. [file 12964_2023_1328_MOESM1_ESM.docx]

**Materials and Methods**

**4.3. Stereotaxic injection**

The animals were anesthetized with sevoflurane (2%–3%) vaporized through a nose cone during surgical procedures. Then, the anesthetized rats were fixed on the stereotaxic apparatus (RWD Life Science Co., Ltd., Shenzhen, CN). Holes were drilled above the CA1 field on two sides of the hippocampus (bregma: anterior/posterior −1.5 mm, medial/lateral ± 1 mm, and dorsal/ventral −1.55 mm). Adeno-associated virus (AAVs) (10^12^ IU/ml, 2 μl) overexpressing cav-1 and control virus were microinfused into the hippocampus via Hamilton microsyringe (Reno, NV, USA). The injection lasted 5 min, and the needle was left in place for 5 min after the injection. We injected AAVs into the hippocampus of mice in week 24. AAVs with cav-1 overexpression were purchased from BrainVTA Co, Ltd. (Wuhan, CN).

**4.6. Western blotting**

Bilateral hippocampi and HT22 cellular samples were collected and homogenized in ice-cold cytoplasmic lysis of RIPA Lysis Buffer with phosphatase inhibitor cocktail (Thermo Fisher Scientific). Then, the homogenate sample was centrifuged at 12000 r/min for 15 mins at 4°C, and the supernatant was collected. An amount of 20-40 μg protein was subjected to 10%-15% SDS polyacrylamide gel electrophoresis, followed by transferring onto polyvinylidene difluoride membranes (PVDF, Millipore, Bedford, MA, USA). The PVDF membranes were blocked in 5% skim milk or BSA for 1h and then incubated overnight at 4 ℃ with corresponding primary antibodies. The proteins were detected with the secondary antibody at room temperature or 1h. Specific bands of target proteins were visualized using the chemiluminescence reagents provided with the ECL kit (Affinity, Shanghai, CN) and the ﬁlm was exposed. The band densities were determined using Image J software and normalized to each internal control. List of antibodies involved in the western blotting is shown in **Supplementary** **Table S1**.

**4.7. Behavioral tests**

The Y maze apparatus was wiped with 75% ethanol between tests to minimize olfactory cues. Spontaneous alternation in the Y maze test was used to assess short-term spatial memory performance. During the test session, each mouse was gently placed at the end of one arm and allowed to move freely through the maze for a single 8-min The series of arm entries was visually recorded by a video camera, and the alternation behavior (defined as successive entries into the three arms without repetitions, in overlapping triplet sets) was analyzed by SMART 3.0 (Panlab, Barcelona, Spain).

MWM apparatus consisted of a rotund plastic pool, a platform, and a camera. The pool was filled with water, which was opacified with titanium dioxide and maintained at 22 ± 1 °C. A 10 cm-diameter escape platform was submerged 1 cm beneath the surface of the water. The camera was connected to a tracking system for recording and analysis. MWM test consisted of platform tests for 5 consecutive days and a probe test on day 5, which were performed by researchers blinded to the grouping. During the training tests, the mice were put into the water-facing groove wall at different quadrants, allowed 90 s to search the hidden platform in the fourth quadrant and then stayed on it for 10 s. On the final day, probe tests were carried out without the platform in the second quadrant for 90s. Swimming speed, escape latency, duration in the target quadrant, and the number of platform crosses were automatically measured by specific software (Taimeng, CN).

**4.8. Hematoxylin-eosin (HE) staining**

After transcardial perfusion, the brains were removed and post-fixed overnight at 4 °C in cold 4% paraformaldehyde and embedded in paraffin. Embedded brain tissues were serially chopped in sections of 4 µm for HE staining. HE staining was examined under a light microscope (Olympus, IX73, Tokyo, Japan) to observe the morphological changes in the hippocampal CA1 region.

**4.11. JC-1 staining**

MMP was evaluated using JC-1 probe (5,5',6,6'-tetrachloro-1,1',3,3'-tetraethyl-benzimidazolylcarbocyanine chloride) according to the manufacturer's instructions (Beyotime Biotechnology). The HT22 cells cultured in small dishes were washed with PBS, then 1ml neurobasal and 1ml JC-1 staining working solution were added and mixed well. After incubating at 37°C for 20 minutes, the supernatant was removed and washed twice with JC-1 staining buffer. A volume of 2ml of cell culture medium was added and the neurons were acquired by an inverted fluorescence microscope (Olympus) with DP74 acquisition setting. The JC-1 aggregates/ monomers ratio was calculated and normalized to the control group.

**4.13. Serum insulin measurement**

Blood samples, which were obtained after the mice fasted for 6 hours, were centrifuged at 2,500 rpm (4 °C) for 5 min and then kept at -80 °C. Samples were quantitatively measured by ELISA (Xinle, Shanghai, CN, xl-Em0483) according to the manufacturer’s instruction. The homeostasis model assessment of insulin resistance (HOMA-IR) was calculated as follows: fasting insulin concentration (mU L−1) × fasting glucose concentration (mg dL−1) × 0.05551)/22.5.

**4.14. Golgi staining**

Mice brains were rapidly removed and immersed in Golgi-Cox solution in the dark at room temperature for two weeks. Then, the brains were cut into 200-μm sections serially. The sections were washed in dd water for 5 min, 2 times, placed in 50% NH4OH for 5 min, and washed again in dd water for 5 min, 2 times. After that, the sections were incubated in 5% sodium thiosulfate for 10 min. Following rinsing in PBS, the sections were dehydrated in graded solutions of ethanol. Images were obtained under a bright field confocal microscope (Leica, Frankfurt, Germany) by z-stack scanning and analyzed by Image J software.

**4.15. Transmission electron microscope (TEM)**

Isolated hippocampi were dissected into 1 mm3 pieces (CA1 region) and then immersed in 2.5% glutaraldehyde in PBS and post-fixed in 1% OsO4 at 4 °C. After being dehydrated, the samples were embedded on a Poly/Bed 812 resin, followed by ultrathin (80 nm) sections with an ultramicrotome. Images were acquired using a transmission electron microscope (Hitachi H-7650, Tokyo, Japan).
