# Supplementary figures and images for: Neuron-targeted overexpression of caveolin-1 alleviates diabetes-associated cognitive dysfunction via regulating mitochondrial fission-mitophagy axis
Source: Cell Commun Signal. 2023 Dec 15;21:357. doi: 10.1186/s12964-023-01328-5 (PMC10722701; doi:10.1186/s12964-023-01328-5)

**Figure S1, The general procedure of this study in vivo.**


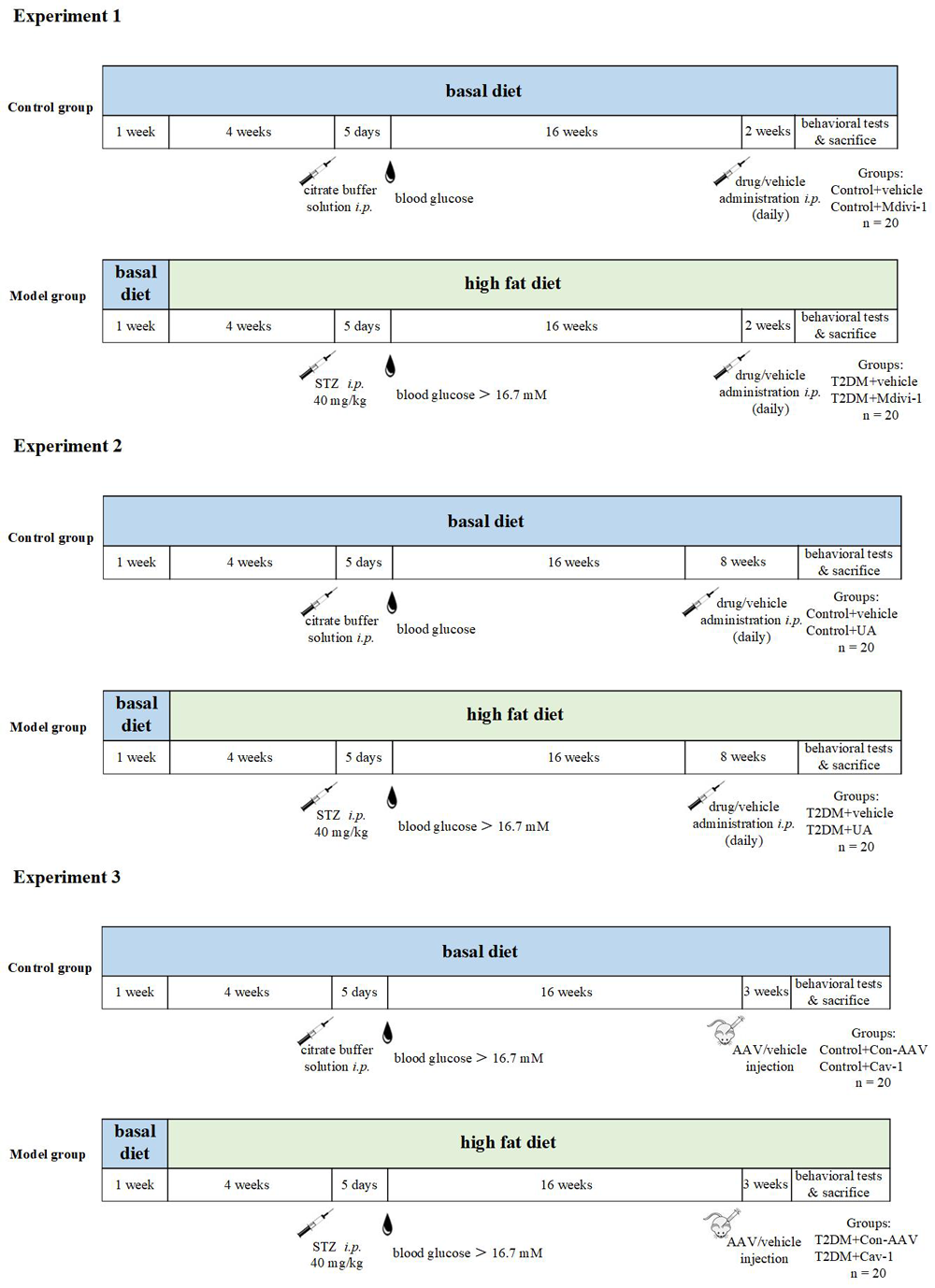

Supplement: Supplementary file 3 — Additional file 2: Figure S1. The general procedure of this study in vivo. [file 12964_2023_1328_MOESM2_ESM.docx]
