# Supplementary material for: Neuron-targeted overexpression of caveolin-1 alleviates diabetes-associated cognitive dysfunction via regulating mitochondrial fission-mitophagy axis
Source: Cell Commun Signal. 2023 Dec 15;21:357. doi: 10.1186/s12964-023-01328-5 (PMC10722701; doi:10.1186/s12964-023-01328-5)
Supplement: Supplementary file 4 — Additional file 3: Figure S2. Mdivi-1 and UA did not exert effects by altering the physiological metabolism. [file 12964_2023_1328_MOESM3_ESM.pdf]

Figure S2, Mdivi-1 and UA did not exert effects by altering the physiological metabolism.

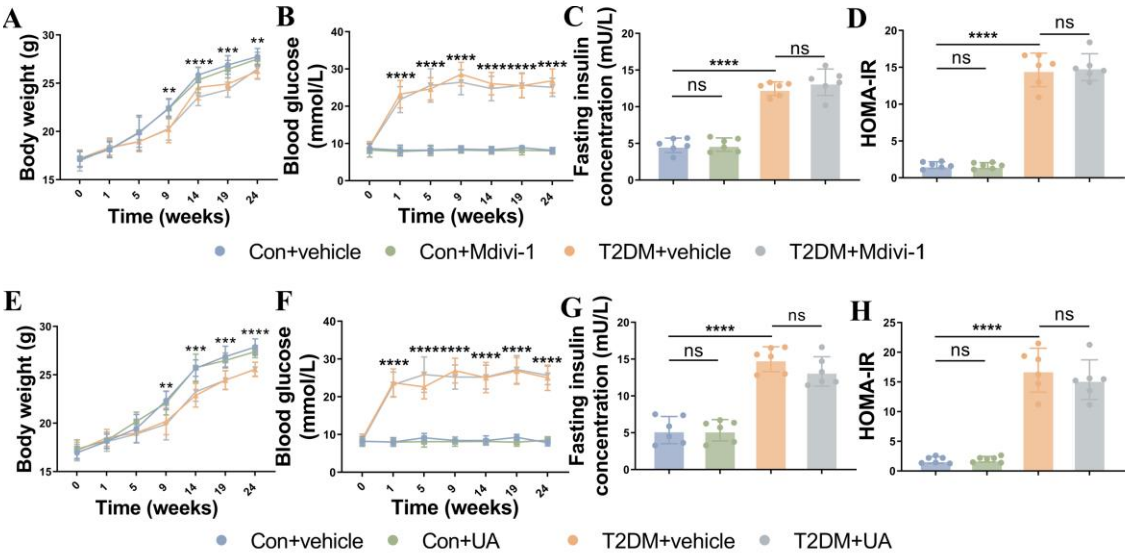

(A-H) Mice body weight (A, E), blood glucose levels (B, F), fasting insulin concentration (C, G), and HOMA-IR (D, H) are monitored and measured (n = 10). The results are represented as mean ± SD. **$P < 0.01$ versus Control+vehicle, ***$P < 0.001$, ****$P < 0.0001$.
