# Supplementary material for: Neuron-targeted overexpression of caveolin-1 alleviates diabetes-associated cognitive dysfunction via regulating mitochondrial fission-mitophagy axis
Source: Cell Commun Signal. 2023 Dec 15;21:357. doi: 10.1186/s12964-023-01328-5 (PMC10722701; doi:10.1186/s12964-023-01328-5)
Supplement: Supplementary file 5 — Additional file 4: Figure S3. Mitophagy is decreased in the HG/Pal-treated HT22 cells. [file 12964_2023_1328_MOESM4_ESM.pdf]

Figure S3, Mitophagy is decreased in the HG/Pal-treated HT22 cells.

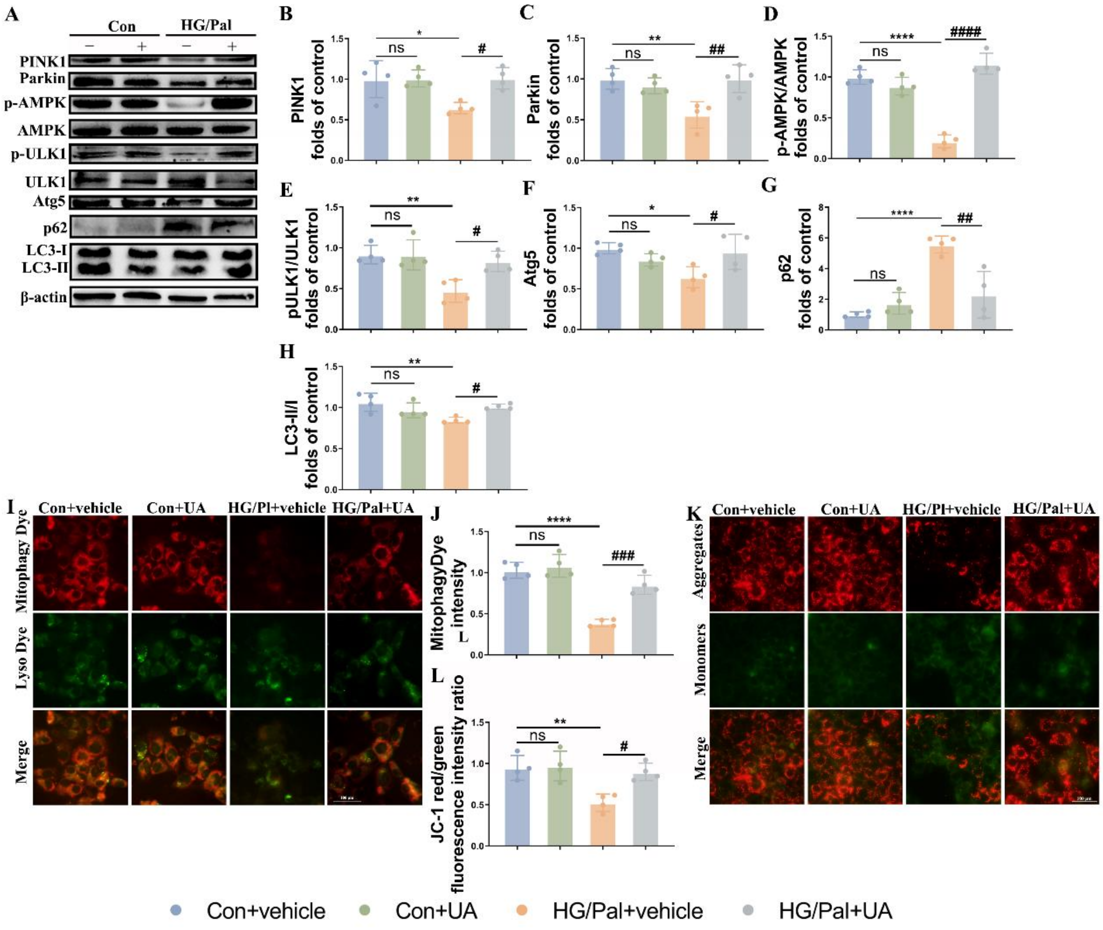

(**A-H**) The proteins expression of PINK1, Parkin, p-AMPK/AMPK, p-ULK1/ULK1, Atg5, p62 and ratio of LC3II/I in the lysates of HT22 cells (n = 4). (**I, J**) Representative mitophagy dye and lyso dye and densitometric analysis of mitophagy dye intensity (n = 4). Scale bar: 100μm. (**K, L**) Mitochondrial membrane potential of HT22 cells was analyzed using JC-1 staining (n = 4). Red and green showed JC-1 aggregates and monomers respectively. Scale bar: 100μm. Values are mean ± SD. **P* < 0.05 versus Control+vehicle, $^{\#}P < 0.05$ versus T2DM+vehicle group, ***P* or $^{\#\#}P < 0.01$, ****P* or $^{\#\#\#}P < 0.001$, *****P* or $^{\#\#\#\#}P < 0.0001$.
