# Supplementary material for: Neuron-targeted overexpression of caveolin-1 alleviates diabetes-associated cognitive dysfunction via regulating mitochondrial fission-mitophagy axis
Source: Cell Commun Signal. 2023 Dec 15;21:357. doi: 10.1186/s12964-023-01328-5 (PMC10722701; doi:10.1186/s12964-023-01328-5)
Supplement: Supplementary file 6 — Additional file 5: Figure S4. Cav-1 overexpression recovers mitochondrial function in T2DM environment. [file 12964_2023_1328_MOESM5_ESM.pdf]

Figure S4, Cav-1 overexpression recovers mitochondrial function in T2DM environment.

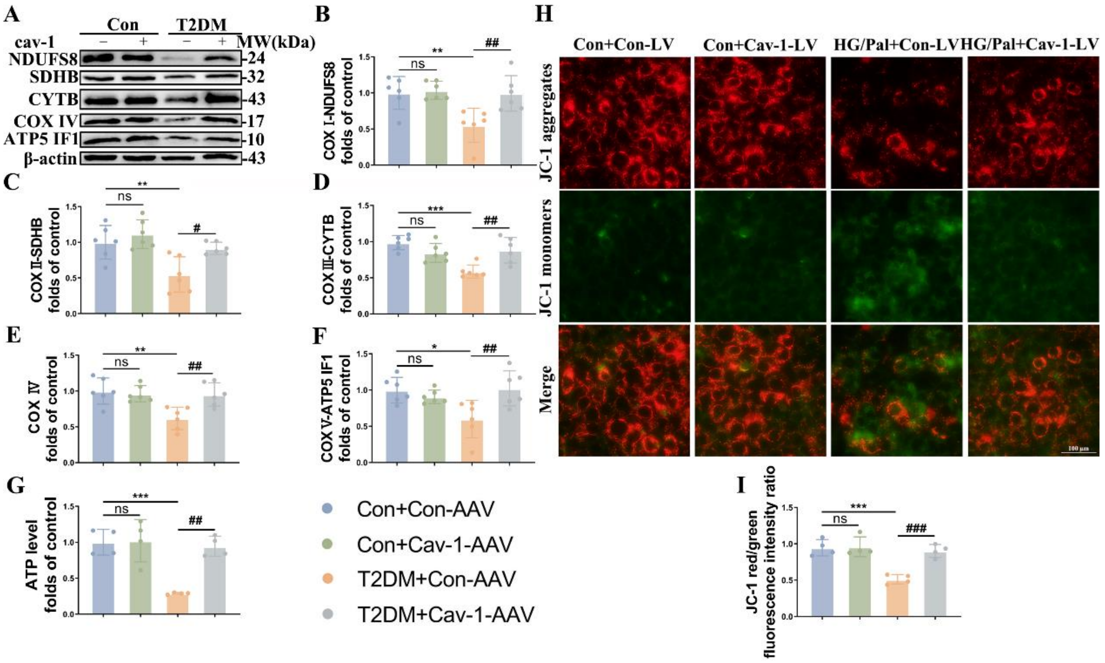

(**A-F**) The proteins expression of complex I-V in the hippocampus of different groups of mice (n = 6 mice per group). (**G**) The level of ATP in the hippocampus of different groups of mice (n = 4 mice per group). (**H, J**) Mitochondrial membrane potential of HT22 cells was analyzed using JC-1 staining (n = 4). Red and green showed JC-1 aggregates and monomers respectively. Scale bar: 100μm. Values are mean ± SD. $^{*}P < 0.05$ versus Control + Con-AAV, $^{\#}P < 0.05$ versus T2DM + Con-AAV group, $^{**}P$ or $^{\#\#}P < 0.01$, $^{***}P$ or $^{\#\#\#}P < 0.001$, $^{****}P < 0.0001$.
