# Supplementary material for: Neuron-targeted overexpression of caveolin-1 alleviates diabetes-associated cognitive dysfunction via regulating mitochondrial fission-mitophagy axis
Source: Cell Commun Signal. 2023 Dec 15;21:357. doi: 10.1186/s12964-023-01328-5 (PMC10722701; doi:10.1186/s12964-023-01328-5)
Supplement: Supplementary file 7 — Additional file 6: Figure S5. [file 12964_2023_1328_MOESM6_ESM.pdf]

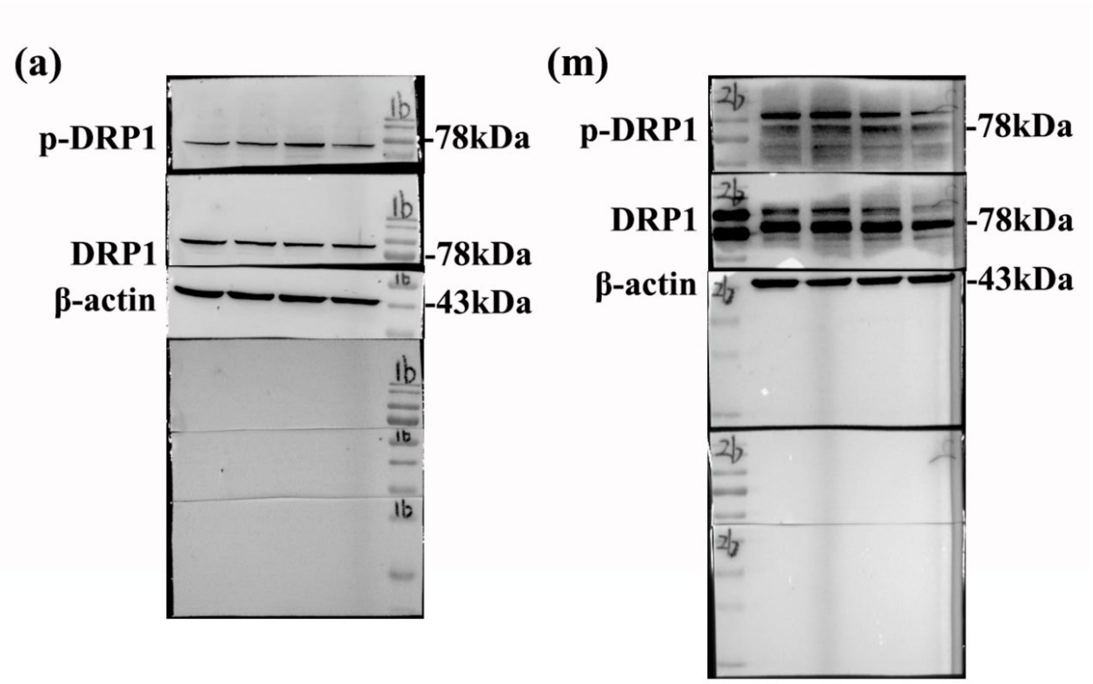

The raw images for the depicted Western blots of Fig. 1(a, m) were used in the manuscript.

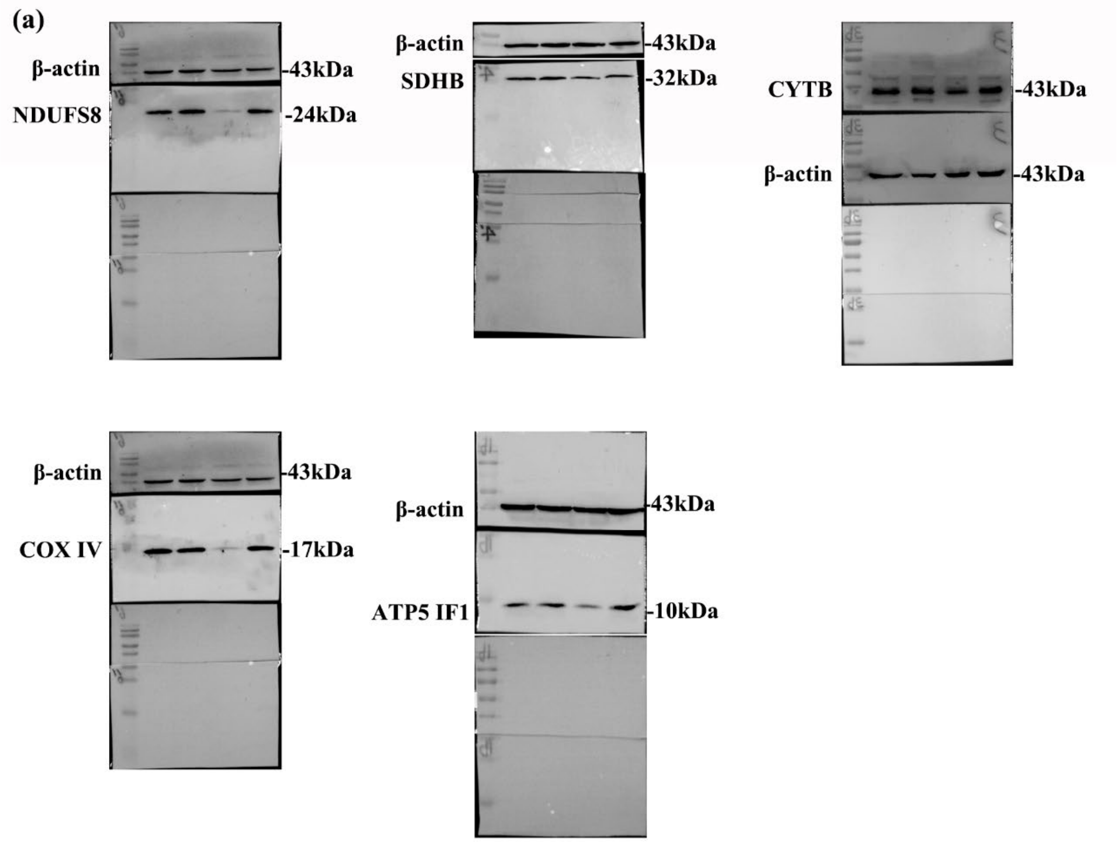

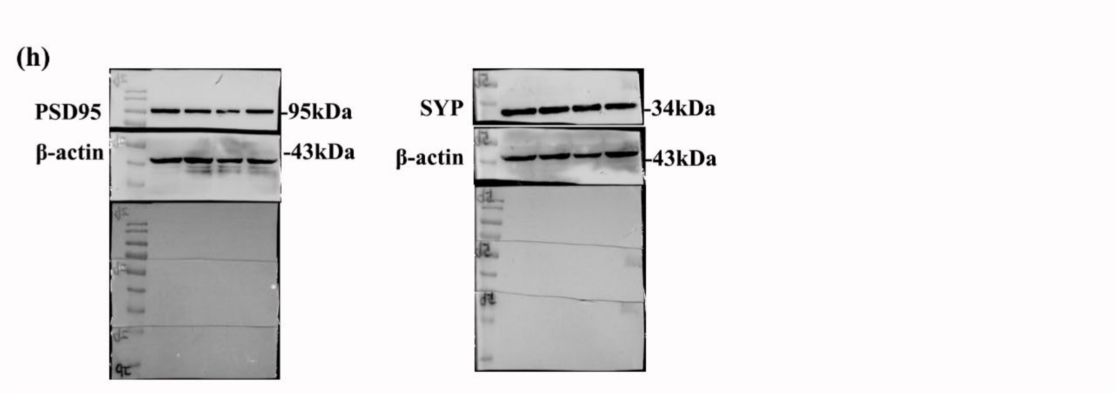

The raw images for the depicted Western blots of Fig. 2(a, h) were used in the manuscript.

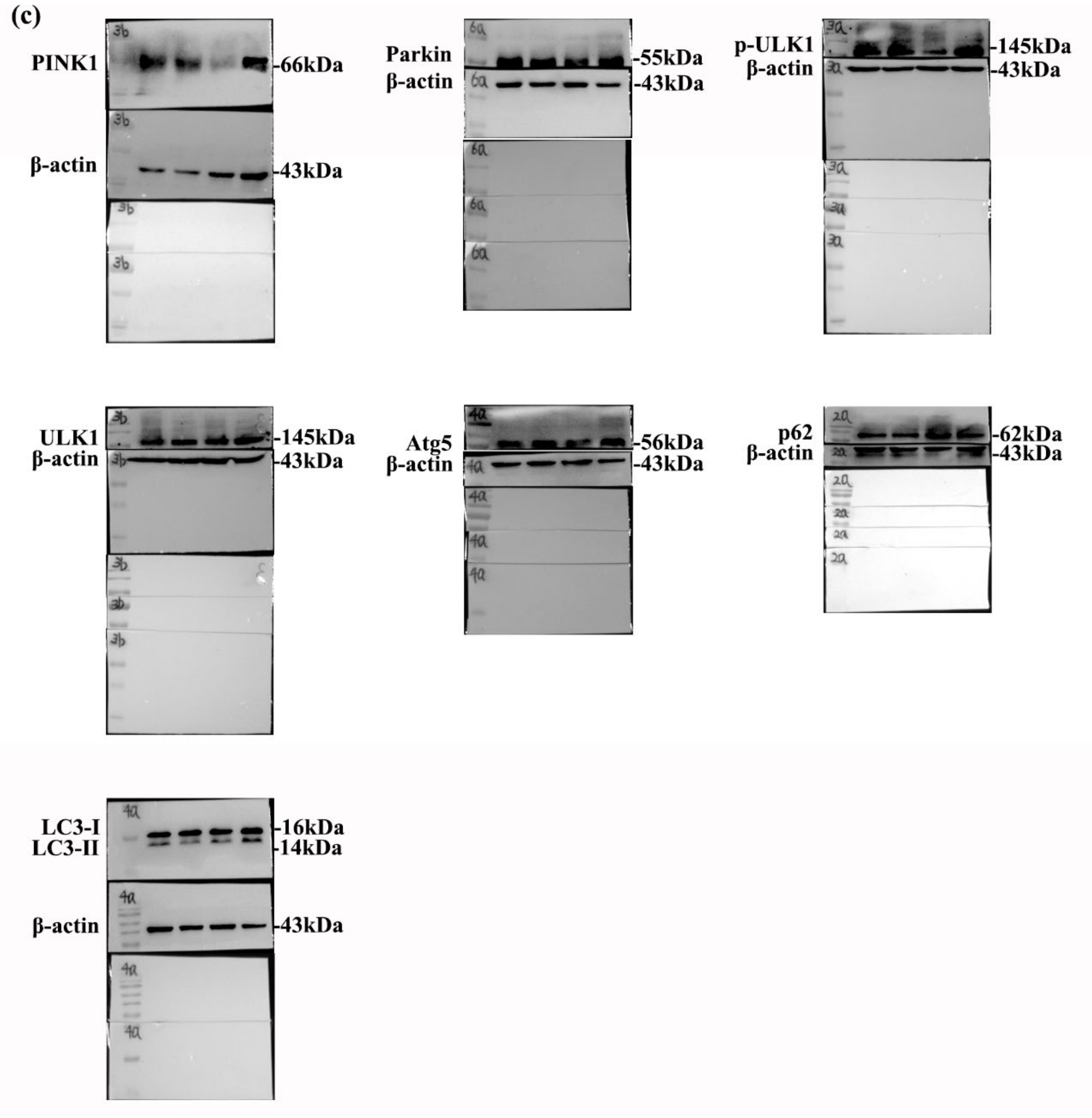

The raw images for the depicted Western blots of Fig. 3(c) were used in the manuscript.

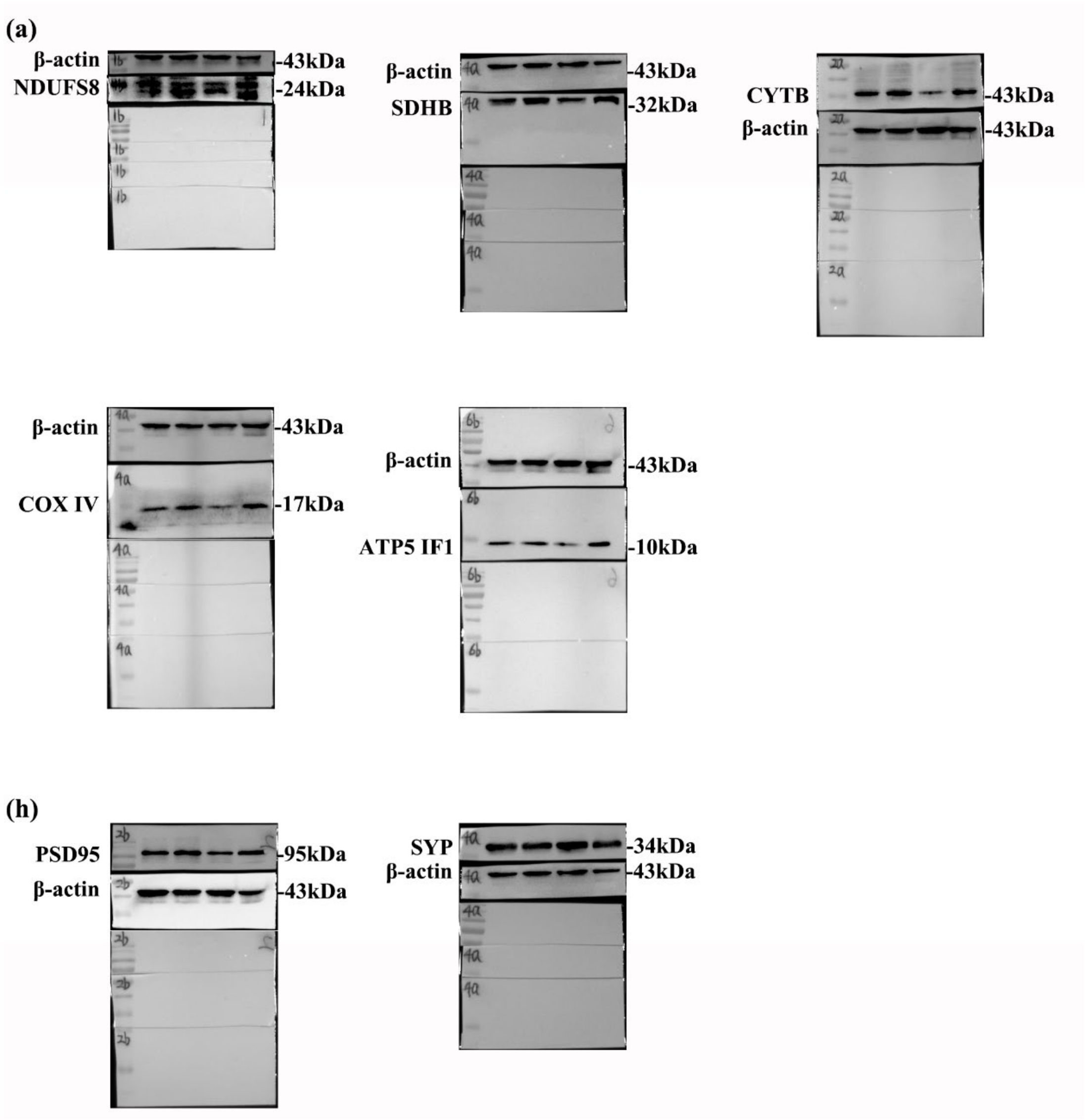

The raw images for the depicted Western blots of Fig. 4(a, h) were used in the manuscript.

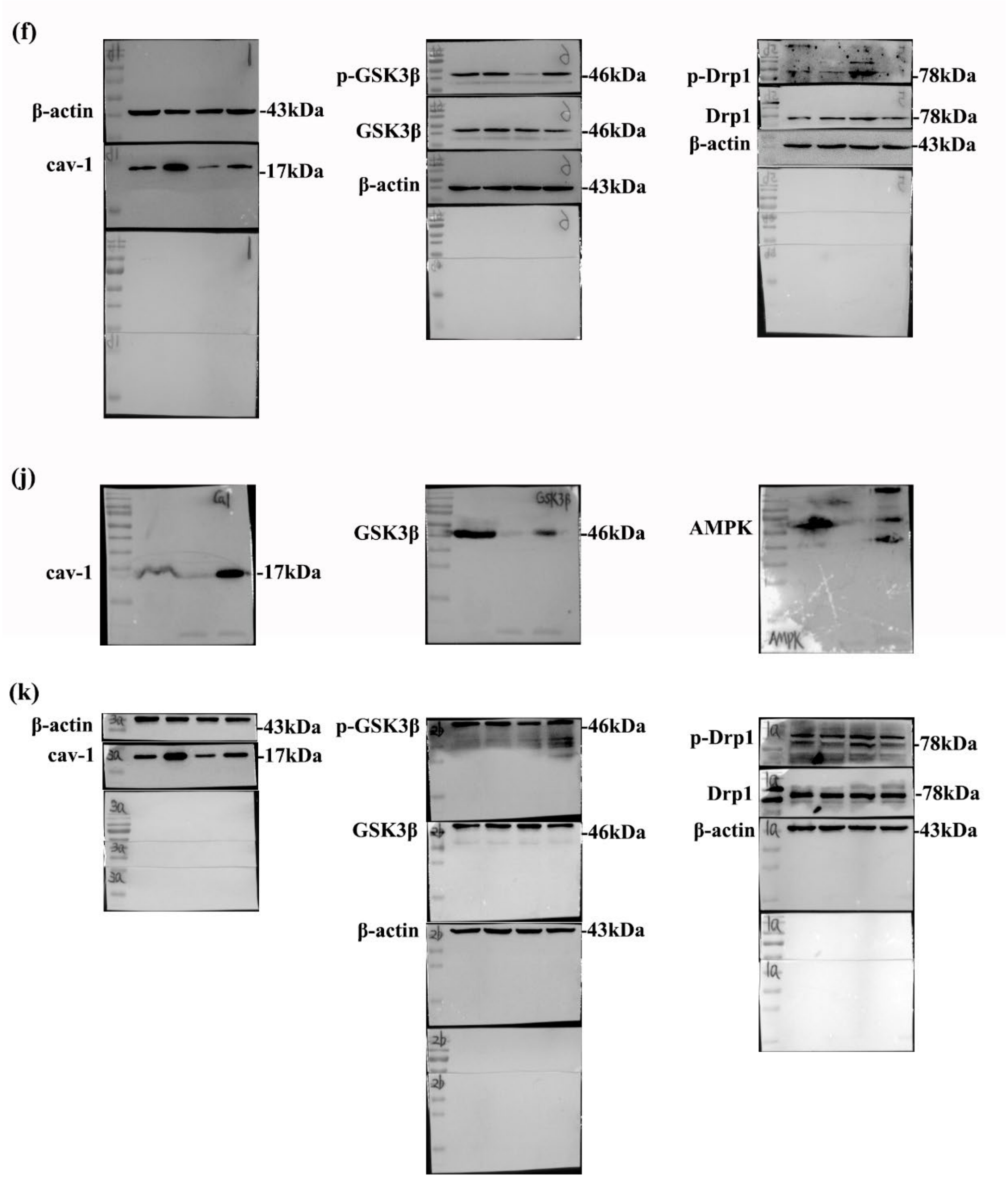

The raw images for the depicted Western blots of Fig. 5(f, j, k) were used in the manuscript.

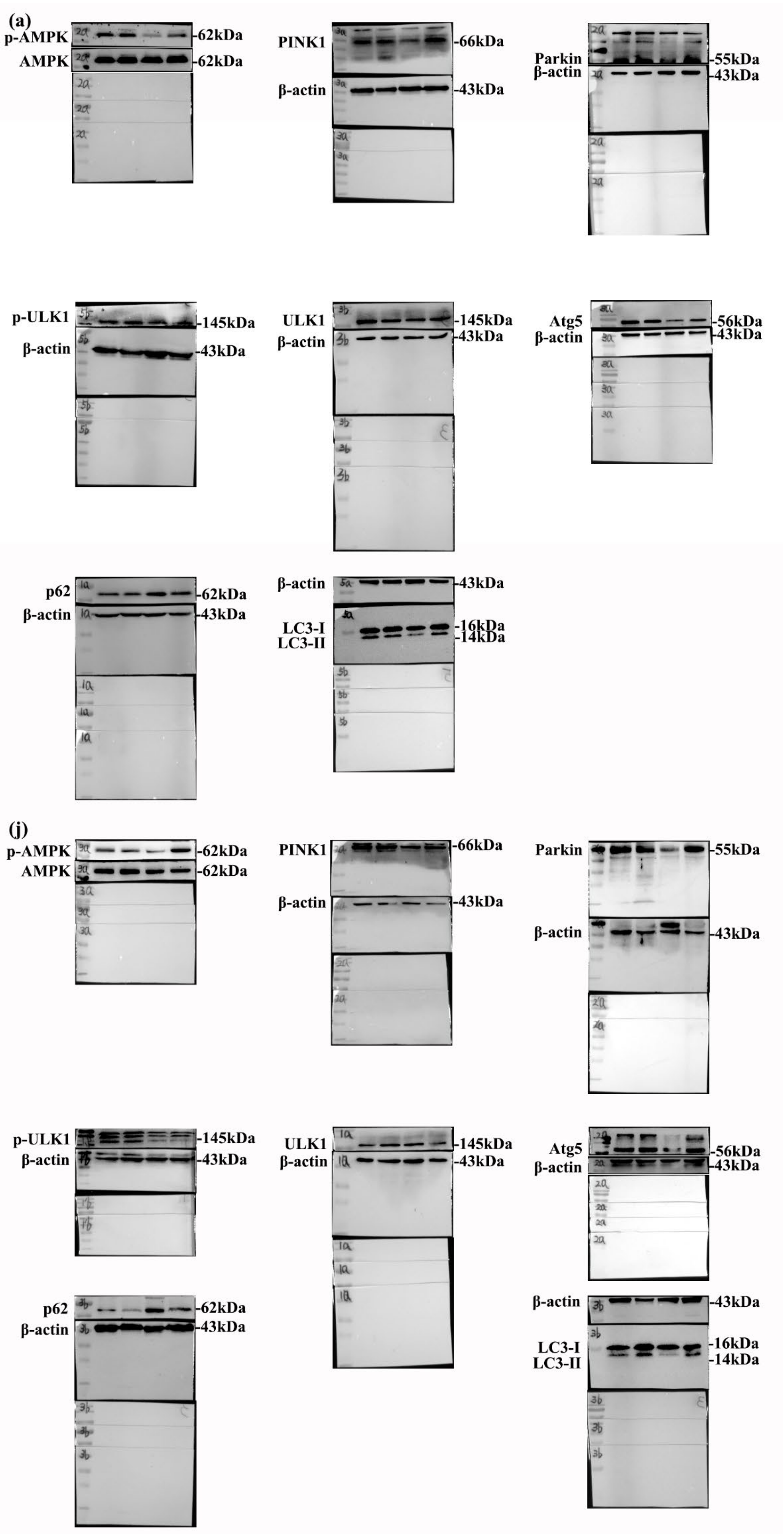
(a)
p-AMPK -62kDa
AMPK -62kDa
PINK1 -66kDa
β-actin -43kDa
Parkin -55kDa
β-actin -43kDa
p-ULK1 -145kDa
β-actin -43kDa
ULK1 -145kDa
β-actin -43kDa
Atg5 -56kDa
β-actin -43kDa
p62 -62kDa
β-actin -43kDa
β-actin -43kDa
LC3-I -16kDa
LC3-II -14kDa
(j)
p-AMPK -62kDa
AMPK -62kDa
PINK1 -66kDa
β-actin -43kDa
Parkin -55kDa
β-actin -43kDa
p-ULK1 -145kDa
β-actin -43kDa
ULK1 -145kDa
β-actin -43kDa
Atg5 -56kDa
β-actin -43kDa
p62 -62kDa
β-actin -43kDa
β-actin -43kDa
LC3-I -16kDa
LC3-II -14kDa

The raw images for the depicted Western blots of Fig. 7(a, j) were used in the manuscript.

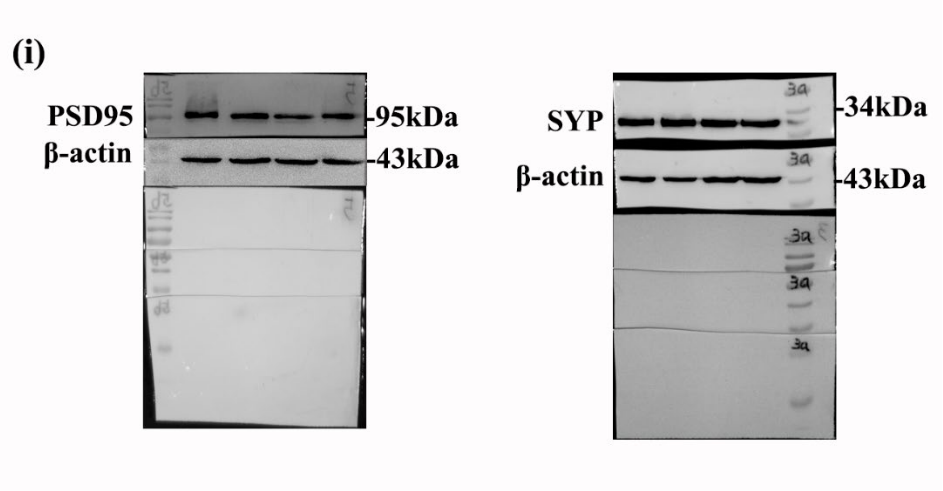

The raw images for the depicted Western blots of Fig. 7(i) were used in the manuscript.

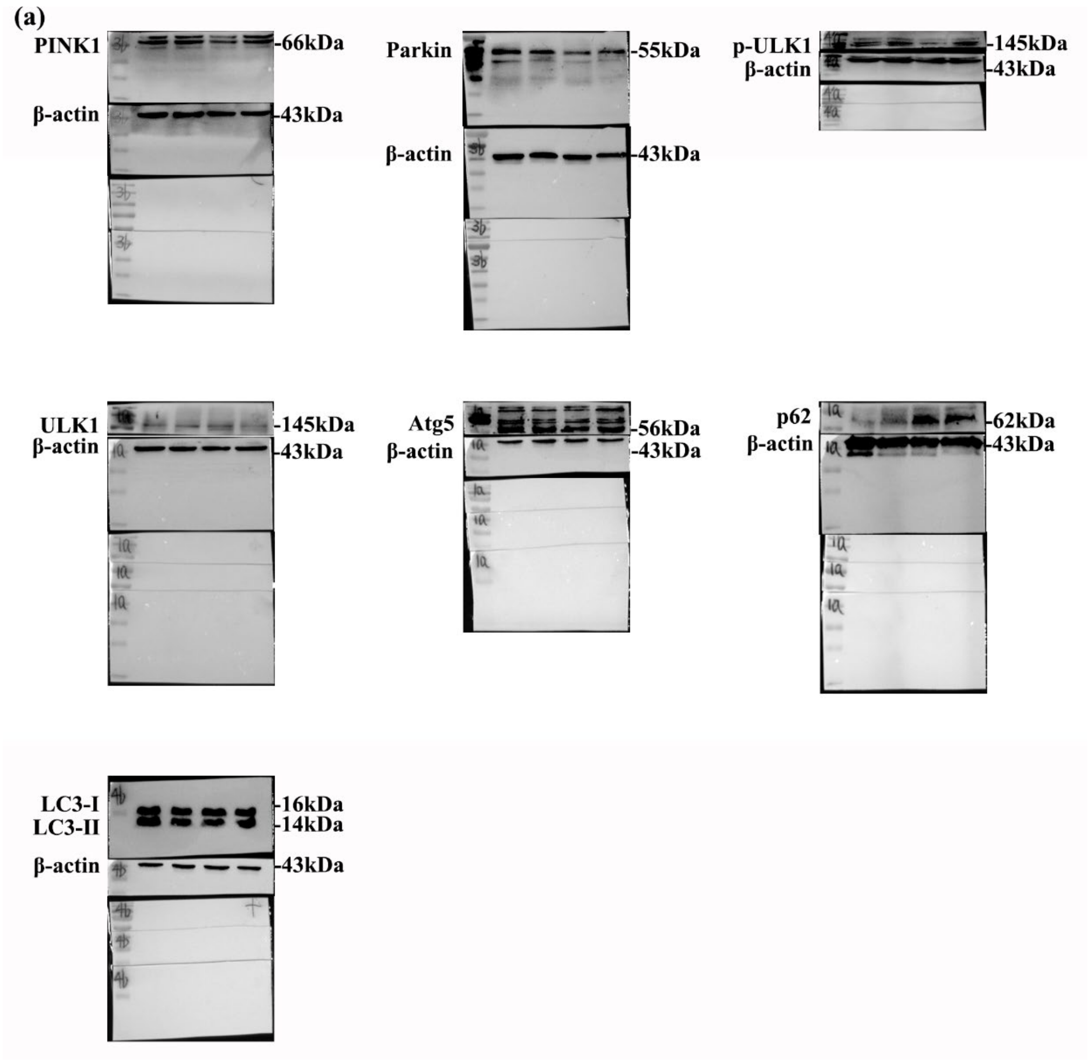

The raw images for the depicted Western blots of Fig. S3(a) were used in the manuscript.

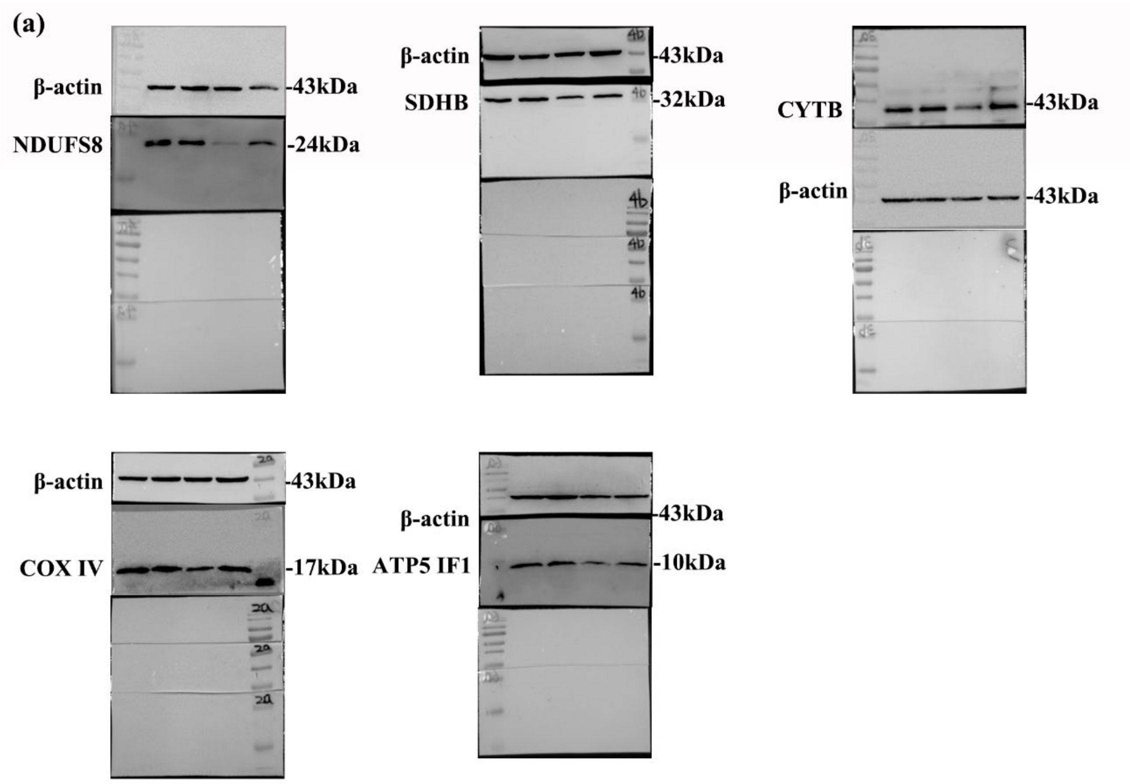

The raw images for the depicted Western blots of Fig. S4(a) were used in the manuscript.
