# Supplementary material for: Neuron-targeted overexpression of caveolin-1 alleviates diabetes-associated cognitive dysfunction via regulating mitochondrial fission-mitophagy axis
Source: Cell Commun Signal. 2023 Dec 15;21:357. doi: 10.1186/s12964-023-01328-5 (PMC10722701; doi:10.1186/s12964-023-01328-5)
Supplement: Supplementary file 8 — Additional file 7: Table S1. The list of primary antibodies. [file 12964_2023_1328_MOESM7_ESM.docx]

| **Antibody name** | **Company** | **Concentration** | **Catalog** |
| --- | --- | --- | --- |
| anti-β-actin | GeneTex | 1:5000 | GTX109639 |
| anti-cav1 | CST | 1:1000 | 3238 |
| anti-Drp1 | Proteintech | 1:1000 | 12957-1-AP |
| anti-pDRP1 (Ser616) | Affinity | 1:1000 | AF8470 |
| anti-NDUFS8 | Proteintech | 1:1000 | 25172-1-AP |
| anti-SDHB | GeneTex | 1:1000 | GTX113833 |
| anti-CYTB | Proteintech | 1:500 | 55090-1-AP |
| anti-COX IV | Proteintech | 1:1000 | 11242-1-AP |
| anti-ATPase IF1 | Abcam | 1:1000 | ab110277 |
| anti-PSD95 | CST | 1:1000 | 3450s |
| anti-SYP | Abcam | 1:1000 | ab32127 |
| PINK1 | Santa Cruz | 1:200 | sc-518052 |
| Parkin | Santa Cruz | 1:200 | sc-32282 |
| anti-ULK1 | CST | 1:1000 | 8054T |
| anti-ULK1 | Santa Cruz | 1:200 | sc-390904 |
| anti-SQSTM1/p62 | CST | 1:1000 | 5114S |
| anti-Atg5 | Novus | 1:1000 | NB-110-53818 |
| anti-LC3B | ABclonal | 1:1000 | A19665 |
| anti-GSK3β | CST | 1:1000 | 12456S |
| anti-GSK-3β (Ser9) | CST | 1:1000 | 5558S |
| anti-pAMPKα (Thr172) | CST, USA | 1:1000 | 2535 |
| anti-AMPKα | CST, USA | 1:1000 | 5381 |

**Table. S1** The list of primary antibodies.
